# Development and validation of circulating protein signatures as diagnostic biomarkers for biliary tract cancer

**DOI:** 10.1016/j.jhepr.2022.100648

**Published:** 2022-12-13

**Authors:** Troels D. Christensen, Emil Maag, Ole Larsen, Claus L. Feltoft, Kaspar René Nielsen, Lars Henrik Jensen, Bonna Leerhøy, Carsten P. Hansen, Inna M. Chen, Dorte L. Nielsen, Julia S. Johansen

**Affiliations:** 1 Deparment of Oncology, Copenhagen University Hospital - Herlev and Gentofte Hospital, Herlev, Denmark; 2 BioXpedia, Aarhus, Denmark; 3 Department of Medicine, Copenhagen University Hospital - Herlev and Gentofte Hospital, Herlev, Denmark; 4 Department of Clinical Immunology, Aalborg University Hospital, Aalborg, Denmark; 5 Department of Oncology, University Hospital of Southern Denmark, Vejle, Denmark; 6 Digestive Disease Center, Copenhagen University Hospital - Bispebjerg and Frederiksberg, Copenhagen, Denmark; 7 Department of Surgery, Copenhagen University Hospital - Rigshospitalet, Copenhagen, Denmark; 8 Department of Clinical Medicine, Faculty of Health and Medical Sciences, University of Copenhagen, Denmark

**Keywords:** biliary tract cancer, cholangiocarcinoma, gall bladder cancer, blood protein assay, multi-biomarker signature, diagnosis, AUC, area under receiver-operating characteristic curve, BBTD, benign biliary tract disease, BP, best point, BTC, biliary tract cancer, CA19-9, carbohydrate antigen 19-9, CAIX, carbonic anhydrase IX, CASP8, caspase 8, CCA, cholangiocarcinoma, CCL, chemokine (C-C motif) ligand, CXCR, C-X-C motif chemokine, dCCA, distal cholangiocarcinoma, EDTA, ethylenediaminetetraacetic acid, GBC, gall bladder cancer, iCCA, intrahepatic cholangiocarcinoma, IL, interleukin, I-O, immuno-oncology, MMP-, matrix metalloproteinase-, NPX, normalized protein expression, pCCA, perihilar cholangiocarcinoma, TME, tumor microenvironment

## Abstract

**Background & Aims:**

Biliary tract cancer (BTC) is associated with a dismal prognosis, partly because it is typically diagnosed late, highlighting the need for diagnostic biomarkers. The purpose of this project was to identify and validate multiprotein signatures that could differentiate patients with BTC from non-cancer controls.

**Methods:**

In this study, we included treatment-naïve patients with BTC, healthy controls, and patients with benign conditions including benign biliary tract disease. Participants were divided into three non-overlapping cohorts: a case-control-based discovery cohort (BTC = 186, controls = 249); a case-control-based validation cohort (validation cohort 1: BTC = 113, controls = 241); and a cohort study-based validation cohort including participants (BTC = 8, controls = 132) referred for diagnostic work-up for suspected cancer (validation cohort 2). Immuno-Oncology (I-O)-related proteins were measured in serum and plasma using a proximity extension assay (Olink Proteomics). Lasso and Ridge regressions were used to generate protein signatures of I-O-related proteins and carbohydrate antigen 19-9 (CA19-9) in the discovery cohort.

**Results:**

Sixteen protein signatures, including 2 to 82 proteins, were generated. All signatures included CA19-9 and chemokine C-C motif ligand 20. Signatures discriminated between patients with BTC *vs.* controls, with AUCs ranging from 0.95 to 0.99 in the discovery cohort and 0.94 to 0.97 in validation cohort 1. In validation cohort 2, AUCs ranged from 0.84 to 0.94. Nine signatures achieved a specificity of 82% to 84% while keeping a sensitivity of 100% in validation cohort 2. All signatures performed better than CA19-9, and signatures including >15 proteins showed the best performance.

**Conclusion:**

The study demonstrated that it is possible to generate protein signatures that can successfully differentiate patients with BTC from non-cancer controls.

**Impact and implications:**

We attempted to find blood sample-based protein profiles that could differentiate patients with biliary tract cancer from those without cancer. Several profiles were found and tested in different groups of patients. The profiles were successful at identifying most patients with biliary tract cancer, pointing towards the utility of multiprotein signatures in this context.

## Highlights

• Several circulating proteins were associated with biliary tract cancer.
• Diagnostic multiprotein signatures were generated and tested.
• The signatures discriminated between individuals with biliary tract cancer and controls.
• Combining several proteins improved diagnostic ability compared to single markers.

## Graphical abstract

**Figure:**
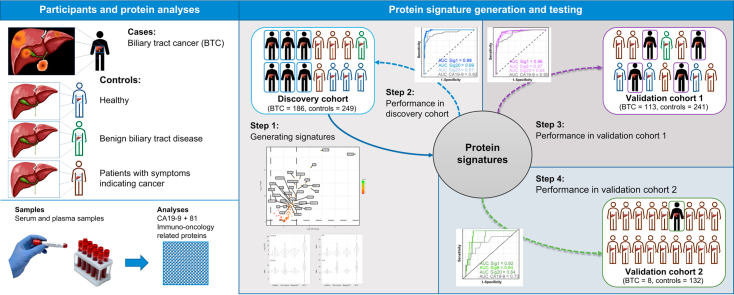

## Introduction

Biliary tract cancer (BTC) is the fifth most common gastrointestinal cancer, with an estimated age-adjusted incidence of about 2–6 per 100,000. It includes both gallbladder cancer (GBC) and cholangiocarcinoma (CCA), which can be further subdivided into distal (dCCA), perihilar (pCCA), and intrahepatic (iCCA).[1], [2], [3], [4], [5], [6]

The overall survival for patients with BTC is less than a year when all stages are included.[7], [8], [9], [10] The poor prognosis is due to the cancer’s aggressive malignant nature, patient comorbidities, and late diagnosis. Patients often experience few, unspecific, or no symptoms at all in the early stages of the disease and are therefore not diagnosed before the disease is advanced. In some patients, the final diagnosis and initiation of treatment can be delayed due to difficulties obtaining usable biopsies. The only potentially curative treatment is surgery, but only a minority of patients are eligible for this treatment due to locally advanced or metastatic disease at the time of diagnosis.[10], [11], [12], [13] Biomarkers that can identify BTC at an early stage are therefore very much needed.

Carbohydrate antigen (CA19-9) is the most widely used biomarker for BTC, but its use as a diagnostic biomarker is limited by low sensitivity and specificity, particularly in patients with early stages of BTC.^14^^,^^15^ Several studies have tried to identify new diagnostic biomarkers; however, none have yet been validated and taken into routine practice.^15^

Chronic inflammation plays a key role in BTC.^11^^,^^16^ This is exemplified by the association between diseases with a high degree of local inflammation, such as primary sclerosing cholangitis and hepatitis, and BTC development.^11^ Activation of inflammatory pathways also affects the tumor microenvironment (TME) and leads to the differentiation of fibroblasts into cancer-associated fibroblasts and the recruitment of macrophages.[16], [17], [18], [19] Multitudes of cytokines including chemokine (C-C motif) ligand (CCL20), epidermal growth factor, hepatocyte growth factor, interleukin (IL)-6, IL-8, and IL-10, and matrix metalloproteases (MMPs), are produced by cancer cells and other cells in the TME, like immune cells and cancer-associated fibroblasts.^17^^,^^20^ The secreted molecules further induce local and systemic inflammation. The complex interplay between cancer cells and stromal cells leads to changing levels of several circulating proteins due to both leakages from the TME and cancer-related inflammation.^17^ Both inflammation-related cytokines, such as IL-6[21], [22], [23] and extracellular matrix modulation-related MMP-7,^24^ have been suggested as diagnostic biomarkers in patients with BTC.

Although a single protein might be used as a diagnostic biomarker,[22], [23], [24], [25] combining several blood proteins in a biomarker signature might yield stronger results.[26], [27], [28] Except for one small study in patients with GBC,^21^ no studies have yet examined the diagnostic use of multiprotein signatures in patients with BTC.

In this study, our aim was to identify and validate circulating multiprotein signatures that could discriminate patients with BTC from non-cancer controls.

## Material and methods

The study was performed and reported in accordance with TRIPOD^29^ guidelines. TRIPOD and CTAT tables are available as supplementary data.

### Patients

The study included 313 treatment-naïve patients with BTC who had been enrolled in two prospective open cohort studies (BIOPAC, CHOCA) and four clinical trials (GI1003, GI1333, GOC-BP, GOX-P) between 2008 and 2020 at three Danish hospitals (Herlev Hospital, Rigshospitalet, and Vejle Hospital). Patients were eligible for the study if they had blood samples collected prior to initiation of treatment and a confirmed diagnosis of BTC, including histological confirmation of malignant disease. Patients with prior treatment for BTC (surgery, radiation, or chemotherapy) were excluded. Likewise, patients with other cancers (except non-melanoma skin cancer and radically treated cancers with no sign of relapse) diagnosed before or within 2 years after diagnosis of BTC were excluded.

A control group consisted of healthy blood donors (n = 180) and patients with benign biliary tract disease (BBTD) (choledocholithiasis, elevated liver enzymes/jaundice, or acute cholangitis) who had an endoscopic retrograde cholangiopancreatography performed (n = 49).

Lastly, a cohort of patients with BTC (n = 8) and controls (n = 394) from a prospective biomarker study (the MICA study) were included. All participants had been referred for a diagnostic work-up due to symptoms, raising suspicion of possible cancer (*e.g*., abdominal pain, fatigue, weight loss). Participants without a cancer diagnosis after a minimum of 2 years’ follow-up were eligible as controls.

None of the control groups included patients with primary sclerosing cholangitis. Thorough descriptions of each study are included in the supplementary materials and methods.

### Ethics

Written informed consent was obtained from all participants. The present biomarker study and original clinical studies all complied with the tenets of the Helsinki Declaration (as revised in 2013) and were approved by the institutional review board of the Regional Danish Ethics Committee: H-3-2014-055 (CHOCA), H-3-2010-053 (GI1003), H-2-2014-026 (GI1333), KA-20060113 (BIOPAC), S-20100051 (GOC-BP), S-20080081 (GOX-P), H-7-2014-011 (MICA), H-15017822 (BBTD cohort).

### Cohorts

**Fig. 1:**
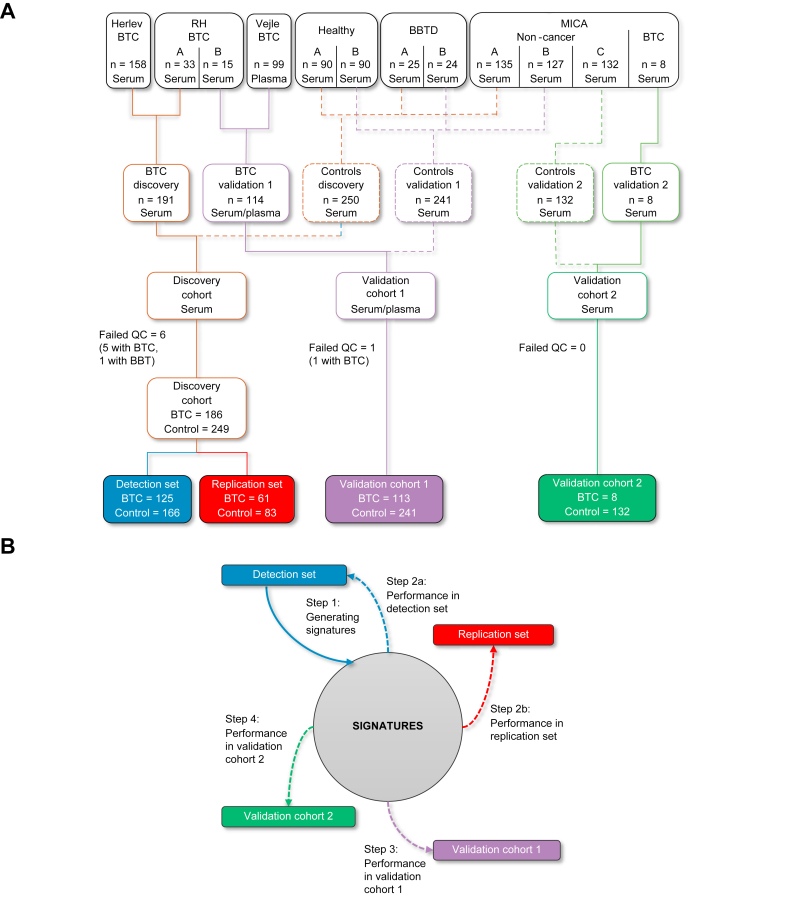
Cohorts used for generation and validation of protein signatures. (A) Consort diagram. (B) Diagram showing all steps in the generation and validation of the protein signatures. BBTD, benign biliary tract disease; BTC, biliary tract cancer; Herlev, Herlev Hospital; MICA, patients referred for diagnostic work-up due to cancer-related symptoms and included in the MICA study; QC, quality control; RH, Rigshospitalet; Vejle, Vejle Hospital.

Patients with BTC and controls were divided into a discovery cohort and two validation cohorts according to a prespecified plan (Fig. 1): The discovery cohort included eligible patients from Herlev Hospital (n = 158) and patients from Rigshospitalet with blood samples collected before January 1, 2016 (n = 33). As controls, half of the patients with BBTD (n = 25) and half of the healthy blood donors (n = 90) were used. Furthermore, all non-cancer controls from the MICA study included prior to March 15, 2017 (n = 135) were included in the discovery cohort. To generate protein signatures, the discovery cohort was divided randomly into a detection set (two-thirds of participants) and a replication set (one-third of participants).

Validation cohort 1 consisted of patients with BTC included at Vejle Hospital (n = 99) and patients from Rigshospitalet included after January 1, 2016 (n = 15). The controls in the cohort were the remaining half of patients with BBTD (n = 24), healthy blood donors (n = 90) and the non-cancer patients included in the MICA study between March 15, 2017 and July 1, 2018 (n = 127).

Validation cohort 2 only included BTC (N = 8) and non-cancer controls included after July 1, 2018 in the prospective MICA study (N = 132). All patients had cancer-related symptoms at referral and at time of blood sample collection, and BTC was diagnosed between 1 and 1,178 days (median 12 days) after the sample was collected. The cohort was designed to simulate a real-world clinical setting in which a biomarker could be used to differentiate patients with BTC from those without cancer.

### Sample characteristics

All samples were collected prior to initiation of therapy. Serum samples were used in most participants; however, serum samples were not available from patients included at Vejle Hospital; in these patients ethylenediaminetetraacetic acid (EDTA) plasma was used.

The serum samples were prepared by centrifuging blood samples within 3 h after blood was drawn at 2,300 G at 4 °C for 10 min, and serum was then aliquoted in Greiner tubes (Cryo.s™ Freezing Tubes, 2 ml, GR-121280, Greiner Bio-One GmbH, Frickenhausen, Germany). The EDTA plasma samples from Vejle Hospital were collected in 9 ml EDTA tubes and centrifuged within 4 h after blood was drawn at 1,486 G at 21 °C for 10 min and transferred to 15 ml cryo tubes. The samples were subsequently stored at −80 °C.

### CA19-9 analyses

Samples from most patients with BTC and all MICA participants were analyzed for CA19-9 prospectively as part of the routine work-up at the same time point as the biobank samples were collected. Samples collected at Rigshospitalet (BIOPAC study) and Herlev Hospital (GI1003, GI1333, CHOCA and MICA studies) were analyzed at the Department of Biochemistry, Herlev Hospital during the same period using the same laboratory equipment and standard operating procedures. Samples from patients with BTC who did not have available results for CA19-9 (n = 14), all patients with BBTD (n = 49), and healthy participants (n = 180) were analyzed in January 2021 and August 2021. Samples were analyzed at the Department of Clinical Biochemistry, Herlev Hospital using Attelica IM CA19-9 (Siemens Healthcare Diagnostics, Duisburg, Germany), a two-step sandwich type chemiluminescent immunometric assay. The intra-laboratory coefficient of variation using internal controls ranged between 5.7 and 6.2%. Elevated CA19-9 was defined as >37 kU/L.

### Protein analyses

A total of 92 proteins were analyzed using the proximity extension assay Immuno-Oncology (I-O) panel from Olink Proteomics, Uppsala, Sweden (www.olink.com). Concentration was measured using an arbitrary unit (normalized protein expression [NPX]) on a Log2 scale. A high NPX value corresponds to a high protein concentration.^30^ The proximity extension assay platform has been widely used for biomarker studies, the method has high accuracy and results obtained using this method correlate well with results obtained using other platforms such as multiple reaction monitoring–mass spectrometry and enzyme-linked immunosorbent assays.[30], [31], [32] This panel was chosen because BTC is characterized by a high degree of inflammation and an immunosuppressive environment,^6^^,^^17^^,^^18^^,^^22^ and the Olink I-O panel has previously been used to generate potential diagnostic protein signatures in pancreatic ductal adenocarcinoma.^26^ The analyses were performed blinded at BioXpedia, Aarhus, Denmark according to the manufacturer’s instructions. The samples from the healthy blood donors were analyzed using the older version of the I-O panel (Proseek® Multiplex Immuno-Oncology, v. 953101; the protein list is available as Table S1). The remaining samples were analyzed using the newer version of the panel (Olink Target 96 Immuno-Oncology, v. 953111/v.953112, Table S2) in three subsequent runs.

Eighty-one proteins were included in the present study. Six proteins were excluded due to changes in assays between I-O panel versions, and five proteins were excluded because more than 90% of values were missing in at least one run. Seven samples were removed due to high internal control deviation or missing data (six samples from the discovery cohort, and one sample from validation study 1). The remaining samples were included in the study (Fig. 1). Detailed description of the protein analysis and quality control is available in the supplementary materials and methods.

### Statistical analysis

No studies have previously described the development of multiprotein predictors in patients with BTC, and exact sample size estimation was not possible. Samples were normalized for any plate effects according to the manufacturer’s recommendations. To compare serum and plasma samples, plasma results were adjusted using a previously identified serum/plasma ratio.^33^

Protein levels were compared with a *t* test or Wilcoxon rank sum test where appropriate. *p* values were adjusted with the Benjamini-Hochberg method. The log2 fold-change was calculated on a linear scale using the geometric mean of each group.

To generate protein signatures, protein levels, including CA19-9 levels, were scaled to unit variance and centered to have a mean equal to zero. Samples with less than 10% missing values were imputed using the function impute.knn from the R-package impute.^34^ The primary set of protein signatures was identified using the 81 proteins + CA19-9. The second set of signatures was generated using CA19-9 + 42 proteins previously identified to have a consistent serum to plasma variation^33^. The signatures were generated using a multi-step strategy based on a similar approach previously employed by our group in a cohort of patients with pancreatic ductal adenocarcinoma.^26^ First, using only the detection set of the discovery cohort, a 500-fold bootstrapped Lasso regression (the R-package glmnet^35^) was performed. For each protein, a proportion score was calculated as the number of times each of the 500 logistic Lasso regression models included that protein as a predictor. The proportion scores were used to generate 21 sets of proteins (signatures). Secondly, signatures were fitted on the detection set using Ridge regression (the R-package glmnet^35^). For each signature, a primary model was fitted to discriminate all patients with BTC from all controls, and secondary models fitted to discriminate between subgroups of patients with BTC and controls. The performance was evaluated using the area under the receiver-operating characteristic curves (AUC). Best point (BP) sensitivity, specificity, positive predictive value and negative predictive value were calculated using the Youden’s Index^36^ and were used as optimal cut-off values. Cut-off values with a sensitivity or specificity above 0.95 were also identified. All signatures were tested with and without age and CA19-9 added as a covariate, and the DeLong test^37^ was used to compare the AUCs in the generated models.

For validation cohort 2, the dataset was sent blinded to the statistician, with no information regarding diagnostic group being given. The statistician employed the signatures using the BP (for all BTC *vs.* all controls) identified in the replication cohort as the threshold for case identification. Afterwards, data were unblinded and performance evaluated. A thorough description of signature generation and evaluation is available in the supplementary materials and methods.

Statistical analyses were performed by a trained bioinformatician (EM) following an analysis plan created prior to initiation of analysis using R (R Core Team (2019. R: A language and environment for statistical computing. R Foundation for Statistical Computing, Vienna, Austria). A two-sided *p* value of 0.05 was considered significant.

## Results

### Patient characteristics

Patient characteristics of the three cohorts are shown in Table 1. Patients with BTC in the discovery cohort more often had iCCA (49.5% *vs.* 33.6%), resectable or locally advanced disease (55.4% *vs.* 30.1%), and performance status 0 or 1 (91.4% *vs.* 79.7%) than those in validation cohort 1. Of the 41 patients with resectable disease, 39 had dCCA, one had GBC (validation cohort 1), and one had iCCA (validation cohort 2). Across all cohorts, patients with BTC had a median age of 67 years *vs.* 63 years for controls, and more often had performance status 1–2 than controls (52.2% *vs.* 15.8%).

**Table 1:** Baseline patient characteristics.

	Discovery cohort	Validation cohort 1	Validation cohort 2
**Biliary tract cancer**			
Number of patients	186	113	8
Female	104 (55.9)	72 (63.7)	3 (37.5)
Male	82 (44.1)	41 (36.3)	5 (62.5)
Age (median [IQR])	67 [58, 71]	66 [57, 73]	77 [74, 83]
iCCA	92 (49.5)	38 (33.6)	5 (62.5)
pCCA	21 (11.3)	18 (15.9)	1 (12.5)
dCCA	40 (21.5)	30 (26.5)	0 (0.0)
GBC	33 (17.7)	18 (15.9)	2 (25.0)
Unknown location	0 (0.0)	9 (8.0)	0 (0.0)
Resectable	27 (14.5)	13 (11.5)	1 (12.5)
Locally advanced	76 (40.9)	21 (18.6)	1 (12.5)
Metastatic disease	83 (44.6)	79 (69.9)	6 (75.0)
PS 0	92 (49.5)	43 (38.1)	5 (62.5)
PS 1	78 (41.9)	47 (41.6)	1 (12.5)
PS 2	5 (2.7)	20 (17.7)	1 (12.5)
PS 3	0 (0.0)	0 (0.0)	1 (12.5)
Unknown PS	11 (5.9)	3 (2.7)	0 (0.0)
CA19-9 (median [range])	188 [1, 297,000]	145 [2, 101,263]	29.50 [1, 3,280]
**Healthy blood donors**			
Number of controls	90	90	0
Female	45 (50.0)	40 (44.4)	—
Male	45 (50.0)	50 (55.6)	—
Age (median [IQR])	62 [57, 65]	63 [56, 65]	—
CA19-9 (median [range])	3.00 [1, 159]	2.00 [1, 35]	
**Benign biliary tract disease**			
Number of patients	24	24	0
Female	17 (70.8)	18 (75.0)	—
Male	7 (29.2)	6 (25.0)	—
Age (median [IQR])	47 [36, 60]	46 [38, 62]	—
PS 0	19 (79.2)	20 (83.3)	—
PS 1	3 (12.5)	2 (8.3)	—
PS 2	2 (8.3)	2 (8.3)	—
PS 3	0 (0.0)	0 (0.0)	—
CA19-9 (median [range])	6.00 [1,34]	6.50 [1,36]	
**MICA study: non-cancer controls**∗			
Number of patients	135	127	132
Female	78 (57.8)	72 (56.7)	80 (60.6)
Male	57 (42.2)	55 (43.3)	52 (39.4)
Age (median [IQR])	60 [50, 70]	65 [54, 72]	68 [57, 73]
PS 0	124 (91.9)	109 (85.8)	100 (75.8)
PS 1	8 (5.9)	18 (14.2)	25 (18.9)
PS 2	2 (1.5)	0 (0.0)	7 (5.3)
PS 3	1 (0.7)	0 (0.0)	0 (0.0)
CA19-9 (median [range])	9 [1, 4440]	9 [1, 315]	1 [1, 723]

CA19-9, carbohydrate antigen 19-9; dCCA, distal cholangiocarcinoma; iCCA, intrahepatic cholangiocarcinoma; pCCA, perihilar cholangiocarcinoma; GBC, gallbladder cancer; PS, performance status.

∗ Participants referred due to symptoms raising suspicion of possible cancer, but no cancer detected after a minimum of 2 years.

### Differences in protein level between patients and controls

**Fig. 2:**
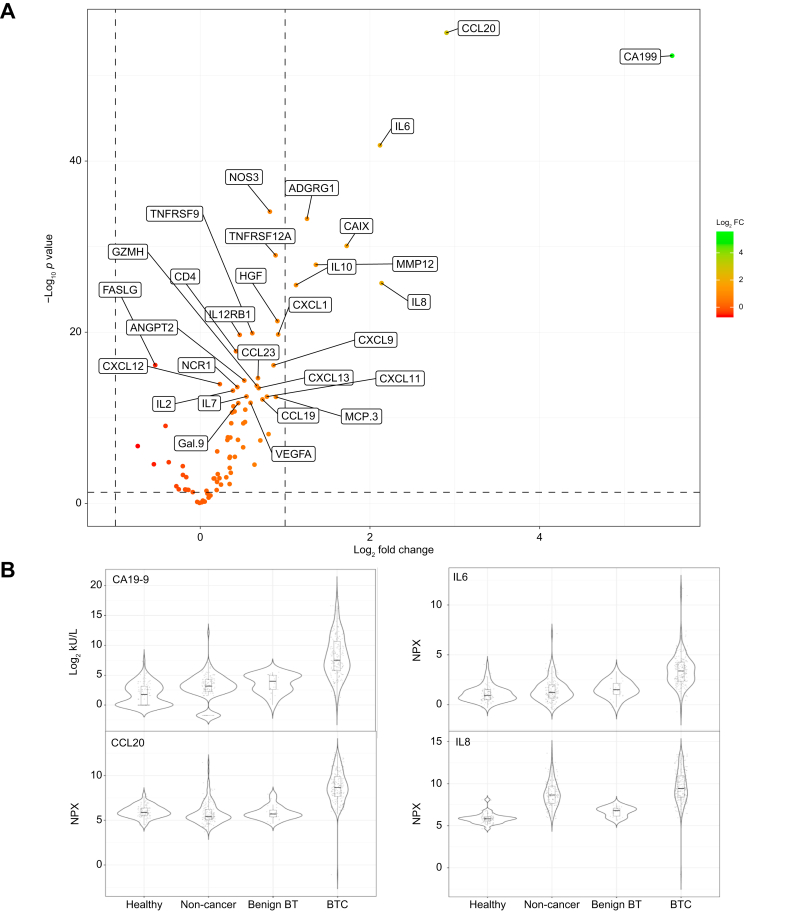
Protein levels in patients with BTC *vs.* controls in the discovery cohort. (A) Volcano plots showing difference (log2 fold-change) in protein level between patients with BTC and all controls for all 82 proteins. Protein levels compared using a *t* test or Wilcoxon rank sum test where appropriate. The 30 proteins with the lowest *p* value are tagged with their name. Vertical dashed lines indicate a log2 fold-change of 1, horizontal dashed line a *p* value of more than 0.05. (B) Boxplots showing difference in protein level between patients with BTC and the three subgroups of controls for the four proteins with largest overall difference. Benign BT, benign biliary tract disease; BTC, biliary tract cancer, CA19-9, carbohydrate antigen 19-9; CCL20, chemokine (C-C motif) ligand 20; Healthy, healthy blood donors; IL, interleukin; non-cancer, non-cancer controls.

**Fig. 3:**
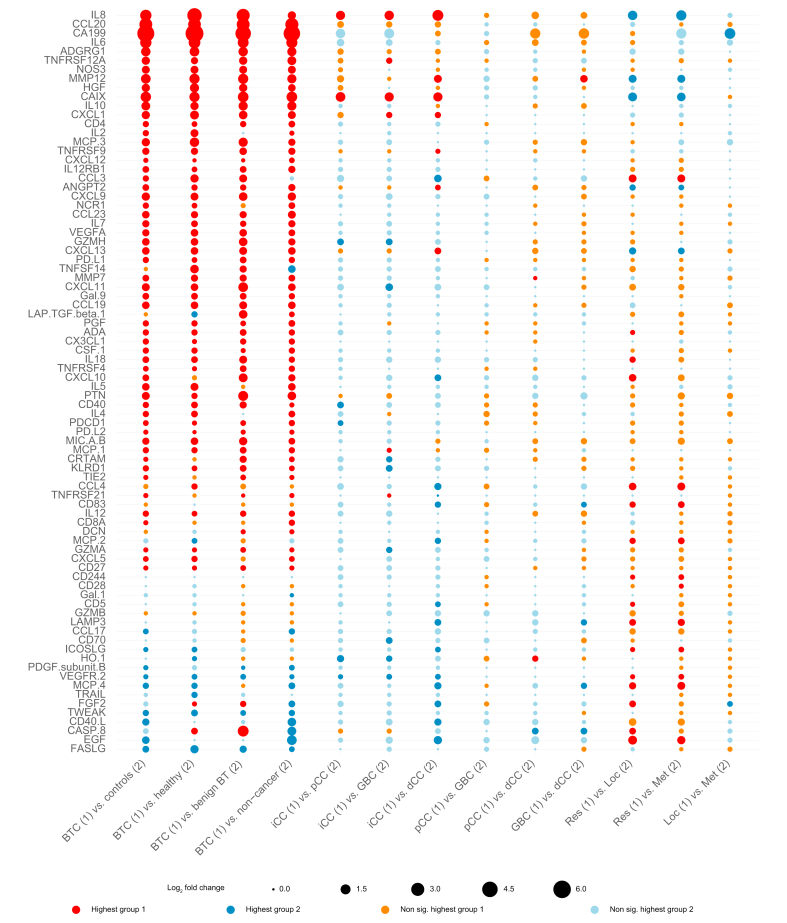
Difference in circulating protein levels between groups of interest in the discovery cohort. Dot plot showing difference in protein level between patients with BTC and subgroups of controls, and between subgroups of patients with BTC. The size of the dot indicates difference in protein level measured as log2 fold-change. For each dot, the color indicates in which group the protein level is highest and whether differences were significant, tested using *t* test or Wilcoxon rank sum test where appropriate. Significant (adjusted *p* value <0.05) differences are dark red (highest in group 1) or navy blue (highest group 2). Non-significant differences are light red (highest group 1) or light blue (highest group 2). Benign BT, benign biliary tract disease controls; BTC, biliary tract cancer; dCCA, distal cholangiocarcinoma; GBC, gallbladder cancer; Healthy, healthy blood donors; iCCA, intrahepatic cholangiocarcinoma; Loc, locally advanced BTC; Met, metastatic BTC; non-cancer, non-cancer controls; Res, resectable BTC.

For the individual proteins, the serum levels were significantly different for CA19-9 and most of the Olink proteins (64 of 81, 79.0%) for BTC *vs.* controls in the discovery cohort. Eight proteins had a log2 fold increase of more than 1: CA19-9, CCL 20, IL-6, IL-8, carbonic anhydrase IX (CAIX), MMP-12, ADGRG1 (adhesion G-protein coupled receptor G1) and IL-10 (Fig. 2). A similar pattern was observed when comparing BTC with subgroups of controls (Fig. 3). Most of the differences between proteins in subgroups of patient with BTC were small; however, IL-8 and CAIX were more than twice as high (log2 fold-change >1) in iCCA than in dCCA, pCCA, or GBC (Fig. 3). Table S3 shows all comparisons made between groups in the discovery cohort. A similar pattern was observed in validation study 1. Notably, CA19-9, CCL20, IL-6, IL-8, CAIX, MMP-12, ADGRG1, and IL-10 were all among the proteins with the highest difference between patients and controls, and again IL-8 and CAIX were notably higher in patients with iCCA than in patients with extrahepatic BTC (Table S4).

### Identification of protein signatures in the discovery cohort

We generated 16 signatures (four signatures were duplicates and were not included in further analysis). The signatures included 2 to 82 proteins. All signatures included CA19-9 and CCL20. The list of proteins, proportion scores, and regression coefficients for all signatures are available in Table S5.

### Signatures’ ability to discriminate BTC from controls in the discovery cohort and validation cohort 1

**Fig. 4:**
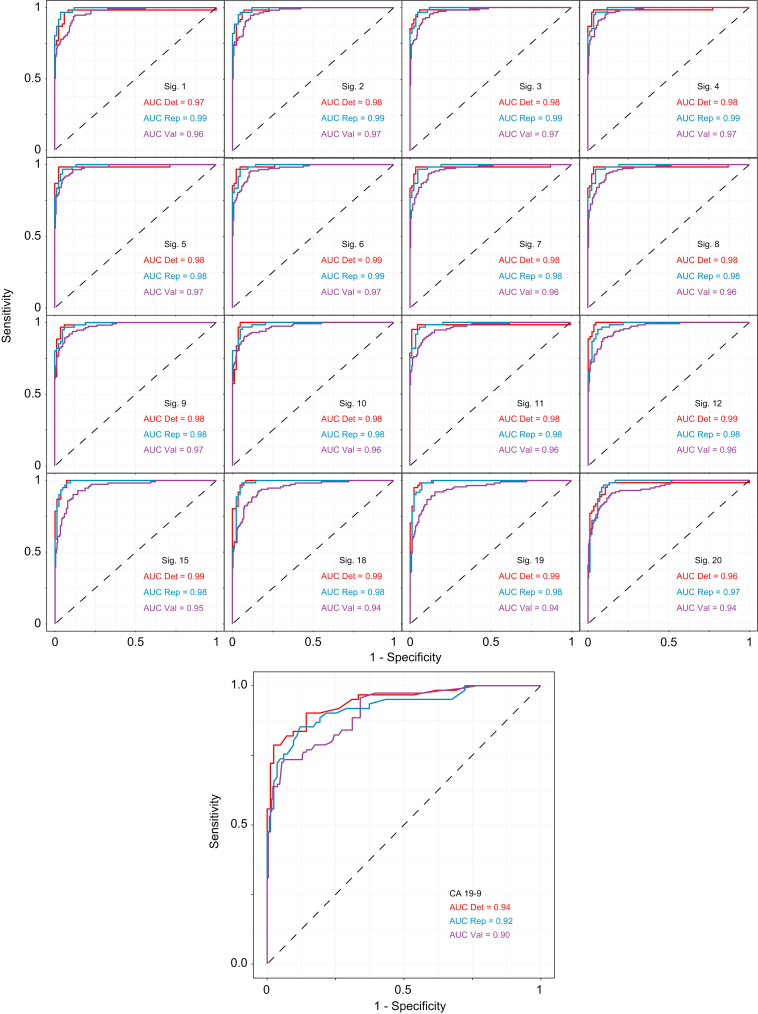
Signatures’ performances for differentiating patients with biliary tract cancer from controls in the discovery cohort and validation cohort 1. Receiver-operating characteristic curve for all signatures and CA19-9. AUC, area under the receiver-operating characteristic curve; CA19-9, carbohydrate antigen 19-9; Det, detection set; Rep, replication set; Sig, signature; and Val, validation cohort 1.

All signatures performed well in the detection and replication set of the discovery cohort (Fig. 4). In the replication cohort, AUCs ranged from 0.97 to 0.99, BP sensitivity from 0.95 to 0.98, and BP specificity from 0.90 to 0.96. All signatures performed better than CA19-9 (AUC = 0.92, BP sensitivity = 0.85, BP specificity = 0.87), and signatures including ≥4 proteins showed equal performance (Table 2 and Table S6).

**Table 2:** Performance of signatures in the discovery cohort and validation cohort 1.

Sig	#	Discovery cohort - detection set				Discovery cohort - replication set				Validation cohort 1			
		AUC	BPse	BPsp	BPt	AUC	BPse	BPsp	BPt	AUC	BPse	BPsp	BPt
1	82	0.97 (0.94–1.00)	0.96 (0.91–1.00)	0.94 (0.88–0.98)	0.42	0.99 (0.98–1.00)	0.96 (0.91–1.00)	0.96 (0.9–1.00)	0.58	0.96 (0.94–0.98)	0.94 (0.89–0.98)	0.87 (0.84–0.92)	0.42
2	61	0.98 (0.97–0.99)	0.98 (0.91–1.00)	0.92 (0.88–0.98)	0.34	0.99 (0.98–1.00)	0.96 (0.91–1.00)	0.95 (0.89–1.00)	0.55	0.97 (0.95–0.98)	0.94 (0.88–0.98)	0.88 (0.85–0.94)	0.45
3	43	0.98 (0.97–1.00)	0.98 (0.91–1.00)	0.94 (0.9–1.00)	0.35	0.99 (0.98–1.00)	0.96 (0.91–1.00)	0.95 (0.89–1.00)	0.5	0.97 (0.95–0.98)	0.92 (0.87–0.98)	0.92 (0.85–0.96)	0.58
4	37	0.98 (0.95–1.00)	0.98 (0.93–1.00)	0.96 (0.92–1.00)	0.4	0.99 (0.98–1.00)	0.96 (0.91–1.00)	0.95 (0.89–1.00)	0.5	0.97 (0.96–0.98)	0.91 (0.88–0.99)	0.93 (0.85–0.96)	0.61
5	32	0.98 (0.96–1.00)	0.98 (0.93–1.00)	0.97 (0.94–1.00)	0.38	0.99 (0.98–1.00)	0.96 (0.91–1.00)	0.95 (0.89–1.00)	0.49	0.97 (0.96–0.99)	0.92 (0.87–0.99)	0.93 (0.86–0.97)	0.59
6	27	0.99 (0.98–1.00)	0.96 (0.93–1.00)	0.97 (0.92–1.00)	0.46	0.99 (0.98–1.00)	0.96 (0.91–1.00)	0.95 (0.89–1.00)	0.55	0.97 (0.95–0.98)	0.95 (0.89–0.99)	0.88 (0.85–0.95)	0.47
7	23	0.98 (0.95–1.00)	0.98 (0.93–1.00)	0.96 (0.91–1.00)	0.42	0.98 (0.97–0.99)	0.96 (0.91–1.00)	0.95 (0.89–1.00)	0.58	0.96 (0.95–0.98)	0.93 (0.85–0.98)	0.88 (0.83–0.95)	0.48
8	19	0.99 (0.97–1.00)	0.98 (0.93–1.00)	0.95 (0.91–1.00)	0.36	0.98 (0.97–0.99)	0.96 (0.91–1.00)	0.95 (0.89–0.98)	0.6	0.96 (0.95–0.98)	0.92 (0.84–0.97)	0.91 (0.85–0.97)	0.58
9	18	0.98 (0.97–1.00)	0.96 (0.93–1.00)	0.96 (0.91–1.00)	0.47	0.98 (0.97–0.99)	0.96 (0.9–1.00)	0.93 (0.89–1.00)	0.56	0.97 (0.95–0.98)	0.89 (0.84–0.97)	0.93 (0.86–0.97)	0.63
10	15	0.98 (0.97–1.00)	1 (0.98–1.00)	0.95 (0.9–0.98)	0.36	0.98 (0.97–0.99)	0.96 (0.91–1.00)	0.93 (0.89–0.98)	0.46	0.96 (0.95–0.98)	0.9 (0.84–0.96)	0.92 (0.87–0.97)	0.59
11	12	0.98 (0.94–1.00)	0.95 (0.91–1.00)	0.98 (0.92–1.00)	0.56	0.98 (0.97–0.99)	0.96 (0.91–1.00)	0.93 (0.89–0.98)	0.47	0.96 (0.94–0.98)	0.9 (0.84–0.97)	0.89 (0.83–0.96)	0.48
12	9	0.99 (0.99–1.00)	1 (0.95–1.00)	0.95 (0.91–1.00)	0.4	0.98 (0.97–0.99)	0.95 (0.88–1.00)	0.93 (0.86–0.98)	0.54	0.96 (0.94–0.97)	0.89 (0.83–0.96)	0.9 (0.82–0.95)	0.47
15	6	0.99 (0.98–1.00)	1 (0.93–1.00)	0.92 (0.88–1.00)	0.36	0.98 (0.97–1.00)	0.98 (0.93–1.00)	0.92 (0.87–1.00)	0.33	0.95 (0.93–0.97)	0.9 (0.84–0.98)	0.88 (0.8–0.94)	0.49
18	5	0.99 (0.98–1.00)	0.96 (0.95–1.00)	0.95 (0.88–0.98)	0.44	0.98 (0.96–1.00)	0.98 (0.93–1.00)	0.93 (0.89–0.98)	0.42	0.94 (0.92–0.96)	0.92 (0.82–0.97)	0.83 (0.79–0.94)	0.4
19	4	0.99 (0.98–1.00)	0.95 (0.91–1.00)	0.97 (0.91–1.00)	0.49	0.98 (0.97–1.00)	0.98 (0.91–1.00)	0.92 (0.87–1.00)	0.41	0.94 (0.91–0.96)	0.92 (0.81–0.95)	0.84 (0.81–0.93)	0.42
20	2	0.96 (0.92–0.99)	0.98 (0.86–1.00)	0.86 (0.82–0.97)	0.29	0.97 (0.95–0.99)	0.96 (0.91–1.00)	0.9 (0.83–0.97)	0.4	0.94 (0.91–0.96)	0.91 (0.8–0.95)	0.85 (0.81–0.95)	0.4
CA 19-9		0.94 (0.9-0.98)	0.78 (0.73–0.96)	0.97 (0.82–1.00)	0.48	0.92 (0.87–0.96)	0.85 (0.72–0.95)	0.87 (0.79–0.98)	0.41	0.9 (0.87–0.94)	0.73 (0.66–0.97)	0.93 (0.66–0.97)	0.57

All values are presented with bootstrapped 95% CIs in parentheses.

AUC, area under receiver-operating characteristic curve; BPse, best point sensitivity; BPsp, best point specificity; BPt, best point threshold; CA19-9, carbohydrate antigen 19-9; Sig, signature.

# Number of proteins.

The signatures’ performances remained high in validation cohort 1 (AUC ≥0.94) (Fig. 4). BP sensitivity ranged from 0.89 to 0.94, and BP specificity from 0.83 to 0.93. All signatures achieved a higher AUC than CA19-9 alone (AUC = 0.90), and signatures including ≥9 proteins all had AUC ≥0.96 (Table 2 and Table S6). A secondary set of 17 signatures including CA19-9 and 1 to 42 proteins with stable serum/plasma variation showed equal performance, with AUCs above 0.94 to 0.97 (Table S7).

Excluding CA19-9 from the signatures decreased their performances, especially in validation cohort 1, where all signatures had a significantly lower AUC after excluding CA19-9 (DeLong test, *p* ≥0.001). After removing CA19-9, the signatures’ AUC remained above 0.95 for signatures 1–10 (including 81–14 proteins), and the lowest AUC was 0.85 for signature 20 (CCL20 alone) in validation cohort 1 (Table S8). Adding age as a variable to the signatures did not significantly improve the AUC of the models (DeLongs test, *p* >0.05) (Data not shown).

### Signature’s ability to discriminate between subgroups

The signatures were also tested for their ability to identify subgroups of patients with BTC divided according to location (iCCA, dCCA, pCCA, GBC) and stage (resectable, locally advanced, and metastatic disease). For most comparisons of controls *vs.* subgroups of BTC, signatures performed better than CA19-9 alone, and signatures that included ≥4 proteins showed similar performance in both the discovery cohort and validation cohort 1 (Table S9). Notably, signatures discriminated well between early stage BTC and controls in both the replication cohort (AUC 0.96–0.98) and validation cohort 1 (AUC 0.92–0.98), and all achieved a higher AUC than CA19-9 alone.

Likewise, the ability of the signatures to identify patients with BTC from subgroups of controls was tested. Signatures had a high AUC in all cohorts when comparing BTC *vs.* healthy (AUC ≥0.96), BTC *vs.* BBTD (AUC ≥0.94), and BTC *vs.* non-cancer controls from the MICA study (AUC ≥0.93). For comparisons between BTC and non-cancer controls, signatures including ≥12 proteins performed best (AUC ≥0.97) (Table S9).

### Blinded prediction in validation cohort 2

Lastly, signatures’ performances were evaluated in a real-world setting in validation cohort 2. All participants in this cohort had been referred to the hospital due to cancer-related symptoms (list of symptoms available in Table S10). Using thresholds defined in the replication cohort, 15 of 16 signatures were able to identify all patients with BTC, giving a sensitivity of 100%. Interestingly, one patient with iCCA and one patient with GBC were diagnosed more than a year after the blood sample was collected (17 months and 38 months, respectively). The specificity for the signatures was 71.2% for the best performing signature (signature 1), and in general the performance decreased the fewer proteins were included. CA19-9 had a sensitivity of 87.5% and specificity of 43.5%. The sensitivity, specificity, negative predictive value and positive predictive value are reported in Table 3.

**Table 3:** Performance of the signatures in validation cohort 2.

Sig	#	Blinded prediction			Unblinded prediction			
		Sensitivity	Specificity	Threshold	AUC	BPse	BPsp	BPt
1	82	1.00	0.71	0.58	0.92 (0.86-0.97)	1.00 (1.00-1.00)	0.82 (0.76-0.94)	0.90
2	61	1.00	0.68	0.55	0.93 (0.88-0.98)	1.00 (1.00-1.00)	0.85 (0.79-0.95)	0.92
3	43	1.00	0.64	0.5	0.93 (0.88-0.98)	1.00 (0.87-1.00)	0.82 (0.76-0.96)	0.88
4	37	1.00	0.61	0.5	0.93 (0.87-0.98)	1.00 (0.87-1.00)	0.80 (0.75-0.95)	0.85
5	32	1.00	0.62	0.49	0.92 (0.87-0.98)	1.00 (0.87-1.00)	0.79 (0.74-0.94)	0.82
6	27	1.00	0.65	0.55	0.94 (0.89-0.98)	1.00 (1.00-1.00)	0.84 (0.79-0.96)	0.88
7	23	1.00	0.69	0.58	0.93 (0.88-0.98)	1.00 (1.00-1.00)	0.83 (0.78-0.94)	0.89
8	19	1.00	0.69	0.6	0.91 (0.86-0.97)	1.00 (1.00-1.00)	0.83 (0.77-0.91)	0.89
9	18	1.00	0.68	0.56	0.91 (0.85-0.96)	1.00 (1.00-1.00)	0.84 (0.78-0.91)	0.89
10	15	1.00	0.64	0.46	0.91 (0.85-0.96)	1.00 (1.00-1.00)	0.84 (0.78-0.91)	0.87
11	12	1.00	0.65	0.47	0.87 (0.80-0.95)	1.00 (0.87-1.00)	0.70 (0.65-0.89)	0.68
12	9	1.00	0.67	0.54	0.87 (0.78-0.95)	1.00 (1.00-1.00)	0.74 (0.67-0.90)	0.69
15	6	1.00	0.48	0.33	0.84 (0.75-0.94)	1.00 (0.87-1.00)	0.64 (0.58-0.89)	0.51
18	5	1.00	0.58	0.42	0.85 (0.76-0.94)	1.00 (0.87-1.00)	0.67 (0.61-0.85)	0.52
19	4	0.88	0.53	0.41	0.85 (0.74-0.96)	0.87 (0.75-1.00)	0.82 (0.50-0.90)	0.74
20	2	1.00	0.52	0.4	0.84 (0.72-0.95)	1.00 (0.75-1.00)	0.61 (0.54-0.94)	0.49
CA19-9		0.88	0.44	0.41	0.73 (0.50-0.95)	0.75 (0.37-1.00)	0.68 (0.45-1.00)	0.59

All values are presented with bootstrapped 95% CIs in parentheses.

AUC, area under receiver-operating characteristic curve; BPse, best point sensitivity; BPsp, best point specificity; BPt, best point threshold; CA19-9, carbohydrate antigen 19-9; Sig., signature.

# Number of proteins.

### Unblinded test in validation cohort 2

**Fig. 5:**
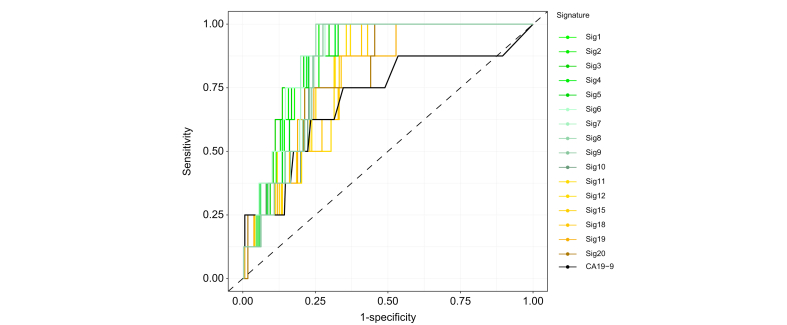
Signatures’ performances for differentiating patients with biliary tract cancer from controls in validation cohort 2. Receiver-operating characteristic curve for all signatures and CA19-9. CA19-9, carbohydrate antigen 19-9; Sig, signature.

After unblinding, the overall ability of the signatures was assessed using receiver-operating characteristic curves, and the optimal threshold for validation cohort 2 was identified (Fig. 5). The AUC was 0.94 for the best performing signature (signature 6: 27 proteins), with a sensitivity of 100% and specificity of 84%. However, eight of the other signatures were able to achieve a specificity of 82–84% while keeping a sensitivity of 100%. All eight signatures included ≥15 proteins. For comparison, CA19-9 had an AUC of 0.73, sensitivity of 75%, and specificity of 68% (Table 3).

## Discussion

Our study demonstrated that it is possible to generate multiprotein blood signatures that can differentiate patients with BTC from non-cancer controls with high sensitivity and reasonable specificity. In general, combining several proteins improved performance compared to single or dual markers, but adding more than 15 proteins to the signatures did not improve the performance significantly in any of our cohorts. Importantly, the results were reproducible in two validation cohorts, including a cohort representing a real-world setting of patients suspected of having cancer.

An approach like ours, where diagnostic blood-based signatures were created using protein panels covering multiple inflammation-related proteins (>10), has only been tested in patients with GBC. Koshiol *et al.* measured the levels of several inflammation-related protein markers in 150 patients with GBC and compared them to levels in patients with gallstones. They created a protein signature based on four proteins (IL-6, IL-16, CCL20, and soluble tumor necrosis factor receptor-1). The score achieved a specificity of 66.6% at a sensitivity of 90% for detecting early GBC ^21^; however, no validation study was performed. Notably, two of the same proteins (CCL20 and IL-6) were included in all our signatures.^21^ Other studies investigating the use of multiprotein signatures as novel biomarkers are limited by a small number of patients with BTC (n <70) and a lack of validation in independent cohorts.[38], [39], [40], [41], [42]

Other blood-based multiplex biomarkers have shown promising performance in detecting patients with BTC. Hu *et al.* performed a study using 359 patients with iCCA and 642 controls. They identified a three-marker model that included miR-21, miR-122, and CA19-9 that achieved an AUC of 0.866 in their validation cohort.^43^ Tumor-associated microparticles and serum metabolites have been used for multi-marker signatures with potential diagnostic potential.^44^^,^^45^ Although the studies are not directly compatible with ours, the protein signatures we identified showed equal or better performance than previous multiplex biomarkers.

We previously found a potential diagnostic signature for patients with pancreatic ductal adenocarcinoma using the Olink I-O panel. The signature included several of the proteins included in our study, including CA19-9, CCL20, caspase-8, and Fas ligand. However, other proteins such as TWEAK (tumor necrosis factor ligand superfamily member 12), NCR1 (natural cytotoxicity triggering receptor) and IL-6, which were widely used in our signatures, were not included in the pancreatic ductal adenocarcinoma signature.^26^

The Olink I-O panel targets proteins related to cancer and the immune system and several of the proteins we studied have previously been found elevated in BTC.^21^^,^^23^ This is in line with our findings, as most of the proteins were elevated in patients with BTC compared with controls. Besides CA19-9, the most prominent was the chemokine CCL20, whose levels in plasma were more than fourfold higher in patients with BTC than in controls in both the discovery and validation cohorts. Both tumor and stromal cells produce CCL20, which, together with its receptor CCR6 (C-C chemokine receptor 6), is involved in leucocyte migration and inflammation. It is associated with treatment resistance and survival, suggesting that the CCL20-CCR6 axis may be a potential treatment target.^20^^,^^46^ Interestingly, CCL20 is expressed more commonly in CCA cells than in normal adjacent tissue.^47^ Two studies found elevated CCL20 in patients with GBC compared to patients with gallstones,^21^^,^^23^ but our study is the first to show and validate an increased plasma level and potential diagnostic use of CCL20 in patients with CCA. We also found that IL-6 and IL-8 were markedly elevated in patients with BTC compared to controls. Both interleukins have previously been associated with BTC outcome, and IL-6 has been suggested as a treatment target.^21^^,^^22^^,^^48^^,^^49^ Several other proteins found to be significantly elevated in patients with BTC have also been found to be elevated in patients with GBC (CCL4, CCL19, CXCL9, CXCL10, CXCL11, CXCL13, IL-10).^21^

The different subtypes of BTC (iCCA, pCCA, dCCA, and GBC) are often characterized as distinct diseases with different molecular alterations, but they also share several similarities, including a high degree of local and systemic inflammation.^6^^,^^17^^,^^18^^,^[21], [22], [23] Notably, the protein signatures performed well in identifying patients with BTC regardless of location, and only minor differences in plasma level were observed between patients with BTC with regard to the individual proteins. The most notable difference was a higher level of CAIX and IL-8 in patients with iCCA than in extrahepatic subtypes. The signatures were also able to identify patients with early stage resectable disease from controls in both the discovery cohort and validation cohort 1. Most patients with resectable disease had dCCA, limiting our ability to evaluate the performance at identifying early stage iCCA, pCCA, or GBC. However, results from validation cohort 2 indicate that our signatures would be able to identify these patients. Here, the signature was able to identify one patient with stage I iCCA and two patients diagnosed with advanced iCCA and GBC more than a year after samples were collected.

The optimal threshold of the signatures for identification of patients with BTC from controls was similar in the discovery cohort and validation cohort 1. Using the threshold identified in validation cohort 2, most signatures achieved a sensitivity of 100%, but the optimal threshold after unblinding was identified as being higher. The explanation could be that validation cohort 2 was imbalanced compared to the other studies, included very few patients with BTC, and had only controls from the MICA study. The optimal threshold was therefore not fully established in this study and should probably be identified for each population of interest.

This study has some limitations. First, the analyzed blood samples from all controls in validation cohort 1 were serum samples, whereas EDTA plasma samples were used from 99 of the 114 patients with BTC, introducing a potential bias. However, the effect of this on the conclusion was probably minor. Similar results were obtained in validation cohort 1 when only proteins with a stable serum/plasma ratio were analyzed. Likewise, signatures performed well in validation cohort 1 when performance was tested in the 15 early stage patients using only serum samples. Second, the control cohort of patients with BBTD had low CA19-9 levels, and the expression of most proteins was more similar to that observed in the two other control groups than that observed in patients with BTC. The reason might be that patients were included doing follow-up at a time when their biliary disease could be in remission. Third, we did not include controls with known risk factors, such as primary sclerosing cholangitis. Therefore, we do not know whether our signatures can distinguish acute BBTD from BTC or identify patients with BTC among high-risk patients with, for example, primary sclerosing cholangitis. Future studies should explore this. Fourth, CA19-9 was not measured using the same laboratory equipment for all patients and controls, introducing a potential bias. However, we do not believe this affected the overall conclusions substantially since most samples were analyzed in the same laboratory (Herlev Hospital), and the signatures achieved a high AUC even after removing CA19-9. Lastly, protein levels of the I-O proteins were only measured using the Olink panel. The reproducibility of our results using other protein detection methods is not known. Furthermore, the Olink panel measures protein level as relative abundance and consequently, thresholds and protein levels observed in this study are not directly transferable to other studies.

The protein signatures were able to identify patients with BTC in individuals referred for a diagnostic work-up due to suspicion of cancer in all three cohorts. In both validation cohorts 1 and 2, signatures including ≥15 proteins showed the best performance. Given the high sensitivity of the models in this setting, protein biomarkers like these could be used to rule out cancer in such a setting or select patients for intensified surveillance programs. The protein signatures might also have a use in patients with a radiologically proven liver tumor, but where a usable biopsy is not possible due to the location of the tumor. Here, such a biomarker could support a diagnosis of BTC and decisions on a treatment strategy that would lead to earlier treatment initiation and a better outcome.

In conclusion, our study identified new potential diagnostic blood-based protein signatures that may help identify patients with BTC from patients without cancer. Although the protein signatures we used need further independent validation, they showed promising performance. The study also validated the use of multiprotein diagnostic cancer biomarkers based on proximity extension assay technology. The next step in the development of a clinically useful biomarker is to investigate how well the signatures can distinguish patients with BTC from patients with other cancers.

## Financial support

The Danish Cancer Society (grant number R218-A13148) provided salary for TDC. Funding for protein analyses and statistical analyses was provided by Beckett-Fonden, Fonden til fremme af klinisk cancerforskning, The A.P. Moller Foundation, and Tømrermester Holms Mindelegat. The funding sources were not involved in the study design; the collection, analysis, interpretation of the data; or writing of the report.

## Authors’ contributions

All authors have accepted responsibility for the entire content of this manuscript and approved its submission. TDC, OL, IMC, DN, and JSJ formulated the initial idea for the project and together with EM drafted the analysis plan. OL, CLF, KRN, LHJ, BL, JSJ and CPH were responsible for the original trials and inclusion of patients. TDC, CLF, KRN, LHJ, BL, and JSJ performed data curation. TDC and JSJ were responsible for identifying suitable samples. EM performed the bioinformatic interpretation and main statistical analysis in collaboration with TDC. TDC performed the initial draft of the manuscript. All authors reviewed and contributed to the final manuscript. All authors read and approved the final manuscript.

## Data availability statement

Data are available upon reasonable request. Data cannot be uploaded online due to regulations by the Danish Data Protection Agency.

## Conflict of interest

The authors declare no conflicts of interest that pertain to this work.

Please refer to the accompanying ICMJE disclosure forms for further details.
