# Supplementary material for: Development and validation of circulating protein signatures as diagnostic biomarkers for biliary tract cancer
Source: JHEP Rep. 2022 Dec 13;5(3):100648. doi: 10.1016/j.jhepr.2022.100648 (PMC9867981; doi:10.1016/j.jhepr.2022.100648)
Supplement: Multimedia component 1 [file mmc1.pdf]

Journal of Hepatology, Volume *5*

Supplemental information

Development and validation of circulating protein signatures as diagnostic biomarkers for biliary tract cancer

Troels D. Christensen, Emil Maag, Ole Larsen, Claus L. Feltoft, Kaspar René Nielsen, Lars Henrik Jensen, Bonna Leerhøy, Carsten P. Hansen, Inna M. Chen, Dorte L. Nielsen, and Julia S. Johansen

Development and validation of circulating protein signatures as diagnostic biomarkers for biliary tract cancer

Troels D. Christensen, Emil Maag, Ole Larsen, Claus L. Feltoft, Kaspar René Nielsen, Lars Henrik Jensen, Bonna Leerhøy, Carsten P. Hansen, Inna M. Chen, Dorte L. Nielsen, Julia S. Johansen

Table of contents

Supplementary materials and methods..2
Supplementary references...8
Supplementary tables..separate excel file

Supplementary materials and methods

Patients

Biliary tract cancer patients from Herlev Hospital, Herlev, Denmark

Patients from Herlev hospital had been included in one of the following studies:

CHOCA study: An ongoing prospective observational open-cohort biomarker study of patients with biliary tract cancer (BTC) treated at the Department of Oncology, Herlev Hospital (Clinical.trials.gov.ID: NCT05184400, Regional Ethics Committee approval number: H-3-2014-055, the Danish Data Protection Agency approval number: BBH-2014-097, I-suite nr. 03260, P-2020-797). All patients referred for treatment for BTC were eligible, and patients were included at initiation of adjuvant, neoadjuvant, or palliative treatment (first-line and later-line treatment). They were treated with different types of chemotherapy or protocolized treatment with targeted monoclonal antibodies, immunotherapy, and/or radiation. The study was initiated in 2015. By October 22, 2020, 220 patients had been included in the CHOCA study; however, only 83 were enrolled at the beginning of 1st-line therapy and had a baseline sample collected before 1st-line therapy. Of these, 62 were eligible for the present study. The remaining were excluded due to the following reasons: prior treatment (surgery and adjuvant therapy) for BTC (N = 15), other cancer at diagnosis or diagnosed within 2 years of BTC diagnosis (N = 4), also included in MICA (N = 1), or patient had combined hepatocellular/cholangiocarcinoma (N =1).

GI1003 study: A phase II trial entitled "Intra-hepatic chemotherapy with oxaliplatin every second week in combination with systemic gemcitabine and capecitabine and in patients with a KRAS-wild type tumor in combination with cetuximab in patient with non-resectable liver metastases from cholangiocarcinoma" [1] (EudraCT: 2010-020188-19, Regional Ethics Committee approval number: H-3-2010-053, the Danish Data Protection Agency approval number HEH.750.24-39). The single-center trial included both cholangiocarcinoma and gallbladder cancer patients, and the inclusion period was from January 2011 to August 2013. In total, 56 patients were included, and baseline samples were available from 50 patients. Of these, 39 were eligible for the present study. The remaining were excluded due to the following reasons: prior treatment (surgery and adjuvant therapy) for BTC (N = 9), other cancer at diagnosis or diagnosed within 2 years of BTC diagnosis (N = 1), or baseline sample collected after initiation of chemotherapy (N = 1).

GI1333 study: A phase II trial entitled: "Randomized 1st-line treatment with gemcitabine, capecitabine, oxaliplatin vs. gemcitabine and cisplatin in patients with cholangiocarcinoma" [2] (EudraCT: 2013-004854-46, Regional Ethics Committee approval number: H-2-2014-026, HEH-

2014-041, the Danish Data Protection Agency approval number: I-Suite 02756BBH). The trial included both cholangiocarcinoma and gallbladder cancer patients. A total of 100 patients were included at 2 sites (Herlev and Vejle Hospital) from July 2014 to November 2017. Eighty-two patients with locally advanced or metastatic BTC were included in the trial at Herlev Hospital. Baseline samples were available from 75 patients, and of these, 57 were eligible for the present study. The remaining were excluded due to the following reasons: prior treatment (surgery and adjuvant therapy) for BTC (N = 14), other cancer at diagnosis or diagnosed within 2 years of BTC diagnosis (N = 1), or baseline sample collected after initiation of chemotherapy (N = 3).

Biliary tract cancer patients from Rigshospitalet, Copenhagen, Denmark

BIOPAC study: The Danish BIOPAC study "BIOmarkers in patients with PAncreatic Cancer (BIOPAC) - can they provide new information of the disease and improve diagnosis and prognosis of the patients?" (ClinicalTrials.gov ID: NCT03311776; www.herlevhospital.dk/BIOPAC/) is a prospective multicenter open cohort study with ongoing enrollment. It has been approved by the Danish Ethics Committee (VEK, j.nr. KA-20060113) and the Danish Data Protection Agency (j.nr. 2012-58-0004; HGH-2015-027; I-Suite j.nr. 03960; and PACTIUS P-2020-834). A subset of the patients was included at the Department of Surgery, Rigshospitalet on suspicion of pancreatic cancer, but were later diagnosed with BTC. As of June 1, 2021, 61 patients with BTC were included in the BIOPAC study prior to a surgical procedure, 48 of whom were eligible for the present study. The remaining patients were excluded due to the following reasons: no available baseline sample (N = 10), prior treatment for BTC (N = 2), or other cancer present at diagnosis or diagnosed within 2 years of BTC diagnosis (N = 1).

Biliary tract cancer patients from Vejle Hospital, Vejle, Denmark

Patients from Vejle hospital were all included in one of the following trials:

GOC-BP study. A phase II trial entitled: "Randomized phase II trial of combination chemotherapy with panitumumab or bevacizumab in patients with inoperable biliary tract cancer without KRAS exon 2 mutations" [3] (Eudract nr. 2010-020385-13, Regional Ethics Committee approval number: S-20100051). Eighty-eight patients with locally advanced or metastatic BTC were randomized to receive chemotherapy (gemcitabine, oxaliplatin, and capecitabine) in combination with either panitumumab or bevacizumab from August 2010 to March 2016. Blood samples were available from 69 patients, and of these 52 were eligible for the present study. The reasons for exclusion were

baseline sample collected after initiation of therapy (N = 7), prior surgery or other treatment for BTC (N = 10). As part of the trial, only EDTA plasma and buffy coat were collected. Therefore, serum samples were not available.

GOX-P study: A phase II trial entitled: "Combined biological treatment and chemotherapy in patients with unresectable cholangiocarcinoma" [4, 5] (Eudract nr. 2008-002367-14, Regional Ethics Committee approval number: S-20080081). The trial was a phase II study in which patients were assigned to treatment based on KRAS exon 2 mutation status. All patients received combination chemotherapy (gemcitabine, oxaliplatin, and capecitabine). Patients with KRAS wild-type tumors also received panitumumab. Patients were included from 2 sites (Vejle, Denmark, Växjö, Sweden). In total, 71 patients were included from October 2008 to 2015, of whom 25 were KRAS exon 2 mutated and 46 were KRAS wild-type. Baseline blood samples were available from 60 patients, and 47 of these patients were eligible for the present study. The reason for exclusion were baseline sample collected after initiation of therapy (N = 4), prior surgery or other treatment for BTC (N = 9). As part of the trial, only EDTA plasma and buffy coat were collected. Therefore, serum samples were not available.

Control cohorts

Controls were included from 1 of the following 3 groups:

MICA Study: The Danish MICA study "New biomarkers in patients referred because of suspected serious illness - are they giving new diagnostic information?" is a prospective open cohort study with ongoing enrollment of patients (older than 18 years) referred to the diagnostic cancer patient pathway at the Diagnostic Outpatient Clinic at Copenhagen University Hospital - Herlev and Gentofte, in the Capital Region of Denmark. The patients are followed from the time of their first visit in the Diagnostic Outpatient Clinic, during evaluation for a cancer diagnosis, and until death. Relevant clinical characteristics of the patients are included in the MICA database. All patients gave written informed consent. The MICA study protocol was approved by the Danish Regional Ethics Committee (H-7-2014-011) and the Danish Data Protection Agency (HEH-2014-105; I-Suite 03330; PACTIUS P-2020-578). In total, 759 patients were included between 2016 and 2019, of whom 8 were diagnosed with BTC (median 12 days (range: 1 – 1178 days) time from date of blood sampling to date of diagnosis. A total of 394 were alive and had no cancer diagnosis after 2 years' follow-up, and they were included as non-cancer controls in the present study. Patients included in the non-cancer control group had the following symptoms at referral: weight loss (50.8%), fatigue

(38.5%), abnormal biochemistry (32.1%), diffuse abdominal pain (23.2%), night sweats (13.8%), pain (9.9%), fever (6.6%), back pain (6.1%), malaise (5.9%). Blood samples were collected at first visit at the Diagnostic Outpatient Clinic.

Benign biliary tract disease (BBTD) cohort: The cohort included 49 patients who had an endoscopic retrograde cholangiopancreatography (ERCP) performed due to BBTD and had been included as controls in a case-control study of post-ERCP pancreatitis at Bispebjerg Hospital, Copenhagen, Denmark [6]. (Regional Ethics Committee approval number: H-15017822). The reasons for ERCP were image-confirmed choledocholithiasis (N = 25), elevated liver enzymes/jaundice (N = 20), or cholangitis (N = 4). Samples were collected during follow-up at a median of 5.2 years (range 1.8 – 7.6 years) after initial ERCP procedure was performed.

Healthy blood donor cohort: Samples were collected from 180 volunteer healthy blood donors at Aalborg University Hospital (Aalborg, Denmark) between January and February 2019.

Protein analysis

Olink immune-Oncology panel

Samples were analyzed for 92 proteins using the proximity extension assay immuno-oncology (I-O) panel from Olink Proteomics, Uppsala, Sweden (www.olink.com). The I-O panel utilizes 92 pairs of antibodies that are linked to DNA oligonucleotides (probes). When a pair of antibodies bind to a target antigen, the probes are brought into proximity. Consequently, the probes are hybridized and extended by a proximity-dependent DNA polymerization event, generating a DNA surrogate marker for the specific antigen (protein). Afterwards, the DNA sequence is quantified by real-time PCR (qPCR). The analyses were performed at BioXpedia, Aarhus, Denmark. BioXpedia was blinded to the study endpoint as no research questions or clinical data were revealed before all samples had been analyzed. For the analyses, serum and EDTA plasma samples were thawed, mixed using a vortex mixer, and centrifuged at 400 g for 1 minute. Then 1 μL of serum or EDTA plasma was transferred to the incubation plate and mixed with the Olink incubation mix and the antibody-probe pairs and analyzed according to the manufacturer's instructions. As recommended by Olink, samples were randomized across assay plates. We also included 2 sample controls (pooled plasma samples), 2 negative controls, and 2 inter-plate control (synthetic samples) on each plate [7]. Additionally, we included 8 bridging samples on all plates used in this project.

Samples were removed if the standard deviation of the internal controls on each plate was above 0.2 NPX, or the deviation from the median value of the controls for each individual sample was above 0.7 NPX. Samples or proteins with more than 10% missing values were removed. Proteins with more than 90% of the value below the limit of detection (LOD) in 1 run were removed.
The samples from the healthy blood donors were analyzed using an older version of the I-O panel (Proseek® Multiplex Immuno-Oncology, v. 953101) in November 2018 and May 2019 as part of a prior study [8]. Later, the manufacturer changed the panel, and the remaining samples were analyzed using a newer version of the panel (Olink Target 96 Immuno-Oncology, v. 953111/v.953112). The samples from patients included in the MICA study were analyzed in October 2019, samples from patients with BTC from Herlev Hospital and patients with BBTD were analyzed in November 2020, and samples from patients included at Vejle Hospital were analyzed in May 2021. A full list of proteins in both panels is available in Supplementary Tables 1 and 2. The following 4 proteins were available only in the old version: IL-21, IL-35, interferon (IFN)-beta, and vascular endothelial growth factor C (VEGFC). These were replaced by the following 4 in the newer version: lymphocyte activation gene 3 protein (LAG3), IL-15, mucin-16 (MUC-16), and killer cell immunoglobulin-like receptor 3DL1 (KIR3DL1). Furthermore, the tumor necrosis factor (TNF) and IFN-gamma assays were markedly improved in the newer version of the panel. Lastly, 5 proteins were removed due to more than 90% of samples being below LOD in at least 1 run (IL-1 alpha, IL-2, IL-13, IL-33, and arginase-1 (ARG1)). Therefore, only 81 proteins were the same in all plates used in the study.

Generation of protein signatures

Samples were normalized for any plate effects using the built-in inter-plate controls according to the manufacturer's recommendations, and the included bridging samples were used for normalization between different version of the I-O panel and plates run at different time points.
Signatures were identified and tested using a multistep strategy. The primary set of protein signatures was identified using the 81 proteins + CA19-9. To test the influence of serum/plasma variabilities on results achieved in validation cohort 1, a second set of signatures was generated using only CA 19-9 + 42 proteins with a constant serum/plasma variation [9].

Step 1: identifying protein signatures in the discovery cohort: The discovery cohort was split randomly into a detection set (2/3 of the data) and a replication set (1/3 of the data). The detection

set was further split randomly into 2 equal parts. A logistic Lasso regression model was fitted on the training set and tested on the test set using the R-package glmnet [10] with alpha = 1 and optimized with the function cv.glmnet using a 10-fold cross validation. This process, including the splitting of the dataset, was repeated 500 times, thus generating 500 different logistic Lasso regression models. For each protein, we calculated the proportion score as the number of times each of the 500 logistic Lasso regression models included that protein as a predictor. The proportion scores were used to generate 21 sets of proteins (signatures). The first set contained proteins with a minimum proportion score of 0 and, thus, contained all the proteins, and the remaining sets were constructed according to incremental steps of the proportion score of 0.05. For sets of proteins that were identical, only the sets with the corresponding highest proportion score were selected, and therefore some of the incremental steps were skipped.

Step 2: fitting signatures on detection set of the discovery cohort: The detection set was split randomly into training and test subsets. For each protein signature, a Ridge regression model was fitted on the training subset of the detection set and tested in the corresponding test subset using the R-package glmnet [10] with alpha = 0 and optimized with the function cv.glmnet using a 10-fold cross validation. The same procedure was carried out with use of the entire detection set as a training set and the entire replication cohort as a test set. For each signature, we fitted a primary model trained to discriminate all BTC from all controls. We also fitted secondary models discriminating between subgroups of BTC (resectable BTC, locally advanced BTC, metastatic BTC, iCC, pCC, dCC, GBC) vs. all controls and all BTC vs. subgroups of controls (patients with BBTD, non-cancer controls, and healthy subjects). Prediction scores were generated for each signature in each cohort. Prediction scores were calculated using NPX and coefficients from each protein as:

$$y = Intercept + NPX.1 * Coefficient.1 + NPX.2 * Coefficient.2 + NPX.X * Coefficient.X$$

$$Prediction\ score = \frac{e^y}{1 + e^y}$$

Step 3: evaluating performance in the discovery cohort: Signature performance was evaluated first in the detection set and afterwards in the replication set. Performance was evaluated using receiver operating characteristic (ROC) curves of prediction scores for each signature and calculated as the area under the ROC curve (AUC). Sensitivity, specificity, positive predictive value and negative predictive value were identified for optimal cut-off calculated using Youden's index

[11]. Likewise, the optimal cut-offs with sensitivity >0.95 and specificity >0.95 were identified. All signatures were tested with and without age and CA19-9 added as a covariates, and the DeLong test [12] was used to compare the AUCs in the generated models. We evaluated each signatures' performance in discriminating all patients with BTC vs. all controls, subgroups of BTC vs. all controls, and all patients with BTC vs. subgroups of controls.

Step 4: evaluating performance in validations cohort 1: Signatures' performance was evaluated in the entire validation cohort 1 using the above-described procedure.

Step 5: evaluating performance in validation cohort 2: The dataset was sent blinded to the bioinformatician who was given no information regarding diagnostic group. The bioinformatician employed the signatures on validation cohort 2 using best point (for all BTC vs. all controls) identified in replication cohort as the threshold for case identification and noted which patients were identified as having BTC. Afterwards, data were unblinded and compared with the prediction. Following this, ROC curves and Youden's index were used to identify optimal threshold for each signature in validation cohort 2.

Supplementary references

[1] Larsen Fo, Hoegdall DTS, Hoegdall E, Nielsen D. Gemcitabine, capecitabine and oxaliplatin with or without cetuximab in advanced biliary tract carcinoma. Acta Oncol 2016;55:382-385.

[2] Markussen A, Jensen LH, Diness LV, Larsen FO. Treatment of patients with advanced biliary tract cancer with either oxaliplatin, gemcitabine, and capecitabine or cisplatin and gemcitabine-a randomized phase II trial. Cancers (Basel) 2020;12:1975.

[3] Amin NEL, Hansen TF, Fernebro E, Ploen J, Eberhard J, Lindebjerg J, et al. Randomized Phase II trial of combination chemotherapy with panitumumab or bevacizumab for patients with inoperable biliary tract cancer without KRAS exon 2 mutations. Int J Cancer 2021;149:119-126.

[4] Jensen LH, Lindebjerg J, Ploen J, Hansen TF, Jakobsen A. Phase II marker-driven trial of panitumumab and chemotherapy in KRAS wild-type biliary tract cancer. Ann Oncol 2012;23:2341-2346.

[5] Jensen L, Andersen R, Byriel L, Fernebro E, Jakobsen A, Lindebjerg J, et al. Phase II study of gemcitabine, oxaliplatin and capecitabine in patients with KRAS exon 2 mutated biliary tract cancers. Acta Oncol 2019;59:1-4.

[6] Leerhøy B, Shabanzadeh DM, Nordholm-Carstensen A, Novovic S, Hansen MB, Jørgensen LN. Pancreatic function following post-endoscopic retrograde cholangiopancreatography pancreatitis: A controlled cohort study with long-term follow-up. United Eur Gastroent J 2018;6:586-594.

[7] Olink User Manual v. 1.9.5: Olink Proteomics, Uppsala Sweden; 2018.

[8] Lindgaard SC, Sztupinszki Z, Maag E, Chen IM, Johansen AZ, Jensen BV, et al. Circulating protein biomarkers for use in pancreatic ductal adenocarcinoma identification. Clinical cancer research : an official journal of the American Association for Cancer Research 2021;27:2592-2603.

[9] Christensen TD, Maag E, Madsen K, Lindgaard SC, Nielsen D, Johansen JS. Determination of temporal reproducibility and variability of cancer biomarkers in serum and EDTA plasma samples using a proximity extension assay. Clin Proteomics 2022;19:39.

[10] Friedman J, Hastie T, Tibshirani R. Regularization paths for generalized linear models via coordinate descent. J Stat Softw 2010;33:1-22.

[11] Ruopp MD, Perkins NJ, Whitcomb BW, Schisterman EF. Youden Index and optimal cut-point estimated from observations affected by a lower limit of detection. Biom J 2008;50:419-430.

[12] DeLong ER, DeLong DM, Clarke-Pearson DL. Comparing the areas under two or more correlated receiver operating characteristic curves: a nonparametric approach. Biometrics 1988;44:837-845.
