# Supplementary material for: Development and validation of circulating protein signatures as diagnostic biomarkers for biliary tract cancer
Source: JHEP Rep. 2022 Dec 13;5(3):100648. doi: 10.1016/j.jhepr.2022.100648 (PMC9867981; doi:10.1016/j.jhepr.2022.100648)
Supplement: Multimedia component 2 [file mmc2.docx]

**Journal of Hepatology**

**CTAT methods**

Tables for a “Complete, Transparent, Accurate and Timely account” (CTAT) are now mandatory for all revised submissions. The aim is to enhance the reproducibility of methods.

- Only include the parts relevant to your study
- Refer to the CTAT in the main text as ‘Supplementary CTAT Table’
- Do not add subheadings
- Add as many rows as needed to include all information
- Only include one item per row

**If the CTAT form is not relevant to your study, please outline the reasons why:**

|  |
| --- |

- 1. **Antibodies**

| **Name** | **Citation** | **Supplier** | **Cat no.** | **Clone no.** |
| --- | --- | --- | --- | --- |
|  |  |  |  |  |

- 1. **Cell lines**

| **Name** | **Citation** | **Supplier** | **Cat no.** | **Passage no.** | **Authentication test method** |
| --- | --- | --- | --- | --- | --- |
|  |  |  |  |  |  |

- 1. **Organisms**

| **Name** | **Citation** | **Supplier** | **Strain** | **Sex** | **Age** | **Overall n number** |
| --- | --- | --- | --- | --- | --- | --- |
|  |  |  |  |  |  |  |

- 1. **Sequence based reagents**

| **Name** | **Sequence** | **Supplier** |
| --- | --- | --- |
|  |  |  |

- 1. **Biological samples**

| **Description** | **Source** | **Identifier** |
| --- | --- | --- |
| **Human blood samples** | **CHOCA study** | **NCT05184400** |
| **Human blood samples** | **GI1003 study** | **EudraCT: 2010-020188-19** |
| **Human blood samples** | **GI1333 study** | **EudraCT: 2013-004854-46** |
| **Human blood samples** | **BIOPAC study** | **NCT03311776** |
| **Human blood samples** | **GOC-BP study** | **2010-020385-13** |
| **Human blood samples** | **GOX-P study** | **2008-002367-14** |
| **Human blood samples** | **MICA Study** |  |
| **Human blood samples** | **Benign biliary tract disease (BBTD) cohort:** |  |
| **Human blood samples** | **Healthy blood donor cohort** |  |

- 1. **Deposited data**

| **Name of repository** | **Identifier** | **Link** |
| --- | --- | --- |
|  |  |  |

- 1. **Software**

| **Software name** | **Manufacturer** | **Version** |
| --- | --- | --- |
|  |  |  |

- 1. **Other (e.g. drugs, proteins, vectors etc.)**

|  |  |  |
| --- | --- | --- |
|  |  |  |

- 1. **Please provide the details of the corresponding methods author for the manuscript:**

| **Troels Dreier Christensen**  **Department of Oncology, Herlev and Gentofte Hospital, Borgmester Ib Juuls Vej 1, DK-2730 Herlev, Denmark**  **E-mail: troels.dreier.christensen.01@regionh.dk, Twitter: DreierTroels**  **Telephone: +45 38681381** |
| --- |

**2.0 Please confirm for randomised controlled trials all versions of the clinical protocol are included in the submission. These will be published online as supplementary information.**

|  |
| --- |
