# Supplementary material for: Development and validation of circulating protein signatures as diagnostic biomarkers for biliary tract cancer
Source: JHEP Rep. 2022 Dec 13;5(3):100648. doi: 10.1016/j.jhepr.2022.100648 (PMC9867981; doi:10.1016/j.jhepr.2022.100648)
Supplement: Multimedia component 5 [file mmc5.pdf]

Research article JHEP|Reports

Development and validation of circulating protein signatures as diagnostic biomarkers for biliary tract cancer

Troels D. Christensen,[1,*] Emil Maag,[2] Ole Larsen,[1] Claus L. Feltoft,[3] Kaspar René Nielsen,[4] Lars Henrik Jensen,[5] Bonna Leerhøy,[6] Carsten P. Hansen,[7] Inna M. Chen,[1] Dorte L. Nielsen,[1,8] Julia S. Johansen[1,3,8]

[1]*Deparment of Oncology, Copenhagen University Hospital - Herlev and Gentofte Hospital, Herlev, Denmark;* [2]*BioXpedia, Aarhus, Denmark;* [3]*Department of Medicine, Copenhagen University Hospital - Herlev and Gentofte Hospital, Herlev, Denmark;* [4]*Department of Clinical Immunology, Aalborg University Hospital, Aalborg, Denmark;* [5]*Department of Oncology, University Hospital of Southern Denmark, Vejle, Denmark;* [6]*Digestive Disease Center, Copenhagen University Hospital - Bispebjerg and Frederiksberg, Copenhagen, Denmark;* [7]*Department of Surgery, Copenhagen University Hospital - Rigshospitalet, Copenhagen, Denmark;* [8]*Department of Clinical Medicine, Faculty of Health and Medical Sciences, University of Copenhagen, Denmark*

JHEP Reports **2023.** https://doi.org/10.1016/j.jhepr.2022.100648

Background & Aims: Biliary tract cancer (BTC) is associated with a dismal prognosis, partly because it is typically diagnosed late, highlighting the need for diagnostic biomarkers. The purpose of this project was to identify and validate multiprotein signatures that could differentiate patients with BTC from non-cancer controls.
Methods: In this study, we included treatment-naïve patients with BTC, healthy controls, and patients with benign conditions including benign biliary tract disease. Participants were divided into three non-overlapping cohorts: a case-control-based discovery cohort (BTC = 186, controls = 249); a case-control-based validation cohort (validation cohort 1: BTC = 113, controls = 241); and a cohort study-based validation cohort including participants (BTC = 8, controls = 132) referred for diagnostic work-up for suspected cancer (validation cohort 2). Immuno-Oncology (I-O)-related proteins were measured in serum and plasma using a proximity extension assay (Olink Proteomics). Lasso and Ridge regressions were used to generate protein signatures of I-O-related proteins and carbohydrate antigen 19-9 (CA19-9) in the discovery cohort.
Results: Sixteen protein signatures, including 2 to 82 proteins, were generated. All signatures included CA19-9 and chemokine C-C motif ligand 20. Signatures discriminated between patients with BTC *vs.* controls, with AUCs ranging from 0.95 to 0.99 in the discovery cohort and 0.94 to 0.97 in validation cohort 1. In validation cohort 2, AUCs ranged from 0.84 to 0.94. Nine signatures achieved a specificity of 82% to 84% while keeping a sensitivity of 100% in validation cohort 2. All signatures performed better than CA19-9, and signatures including >15 proteins showed the best performance.
Conclusion: The study demonstrated that it is possible to generate protein signatures that can successfully differentiate patients with BTC from non-cancer controls.
Impact and implications: We attempted to find blood sample-based protein profiles that could differentiate patients with biliary tract cancer from those without cancer. Several profiles were found and tested in different groups of patients. The profiles were successful at identifying most patients with biliary tract cancer, pointing towards the utility of multiprotein signatures in this context.
© 2022 The Author(s). Published by Elsevier B.V. on behalf of European Association for the Study of the Liver (EASL). This is an open access article under the CC BY license (http://creativecommons.org/licenses/by/4.0/).

Introduction

Biliary tract cancer (BTC) is the fifth most common gastrointestinal cancer, with an estimated age-adjusted incidence of about 2–6 per 100,000. It includes both gallbladder cancer (GBC) and cholangiocarcinoma (CCA), which can be further subdivided into distal (dCCA), perihilar (pCCA), and intrahepatic (iCCA).[1–6]

The overall survival for patients with BTC is less than a year when all stages are included.[7–10] The poor prognosis is due to the cancer's aggressive malignant nature, patient comorbidities, and late diagnosis. Patients often experience few, unspecific, or no symptoms at all in the early stages of the disease and are therefore not diagnosed before the disease is advanced. In some patients, the final diagnosis and initiation of treatment can be delayed due to difficulties obtaining usable biopsies. The only potentially curative treatment is surgery, but only a minority of patients are eligible for this treatment due to locally advanced or metastatic disease at the time of diagnosis.[10–13] Biomarkers that can identify BTC at an early stage are therefore very much needed.

Carbohydrate antigen (CA19-9) is the most widely used biomarker for BTC, but its use as a diagnostic biomarker is limited by low sensitivity and specificity, particularly in patients with early stages of BTC.[14,15] Several studies have tried to identify new diagnostic biomarkers; however, none have yet been validated and taken into routine practice.[15]

Chronic inflammation plays a key role in BTC.[11,16] This is exemplified by the association between diseases with a high degree of local inflammation, such as primary sclerosing cholangitis

Keywords: biliary tract cancer; cholangiocarcinoma; gall bladder cancer; blood protein assay; multi-biomarker signature; diagnosis.
Received 3 October 2022; received in revised form 24 November 2022; accepted 1 December 2022; available online 13 December 2022
* Corresponding author. Address: Department of Oncology, Herlev and Gentofte Hospital, Borgmester Ib Juuls Vej 1, DK-2730 Herlev, Denmark; Tel.: +45 38681381.
E-mail address: troels.dreier.christensen.01@regionh.dk (T.D. Christensen).

ELSEVIER

EASL The Home of Hepatology

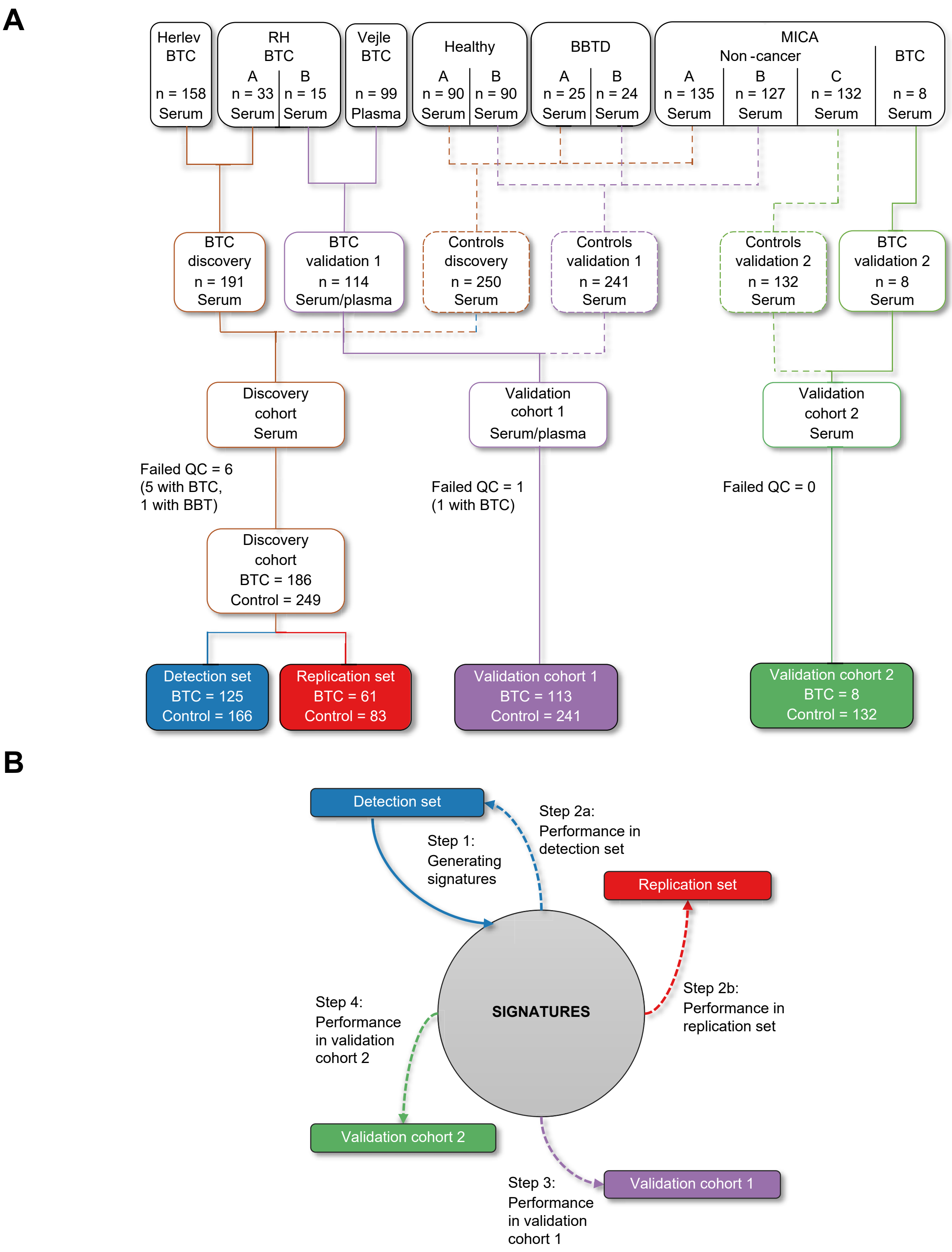

Fig. 1. Cohorts used for generation and validation of protein signatures. (A) Consort diagram. (B) Diagram showing all steps in the generation and validation of the protein signatures. BBTD, benign biliary tract disease; BTC, biliary tract cancer; Herlev, Herlev Hospital; MICA, patients referred for diagnostic work-up due to cancer-related symptoms and included in the MICA study; QC, quality control; RH, Rigshospitalet; Vejle, Vejle Hospital.

and hepatitis, and BTC development.[11] Activation of inflammatory pathways also affects the tumor microenvironment (TME) and leads to the differentiation of fibroblasts into cancer-associated fibroblasts and the recruitment of macrophages.[16–19] Multitudes of cytokines including chemokine (C-C motif) ligand (CCL20), epidermal growth factor, hepatocyte growth factor, interleukin (IL)-6, IL-8, and IL-10, and matrix metalloproteases (MMPs), are produced by cancer cells and other cells in the TME, like immune cells and cancer-associated fibroblasts.[17,20] The secreted molecules further induce local and systemic inflammation. The complex interplay between cancer cells and stromal cells leads to changing levels of several circulating proteins due to both leakages from the TME and cancer-related inflammation.[17] Both inflammation-related cytokines, such as IL-6[21–23] and extracellular matrix modulation-related MMP-7,[24] have been suggested as diagnostic biomarkers in patients with BTC.

Although a single protein might be used as a diagnostic biomarker,[22–25] combining several blood proteins in a biomarker signature might yield stronger results.[26–28] Except for one small study in patients with GBC,[21] no studies have yet examined the diagnostic use of multiprotein signatures in patients with BTC.

In this study, our aim was to identify and validate circulating multiprotein signatures that could discriminate patients with BTC from non-cancer controls.

Material and methods

The study was performed and reported in accordance with TRIPOD[29] guidelines. TRIPOD and CTAT tables are available as supplementary data.

Patients

The study included 313 treatment-naïve patients with BTC who had been enrolled in two prospective open cohort studies (BIOPAC, CHOCA) and four clinical trials (GI1003, GI1333, GOC-BP, GOX-P) between 2008 and 2020 at three Danish hospitals (Herlev Hospital, Rigshospitalet, and Vejle Hospital). Patients were eligible for the study if they had blood samples collected prior to initiation of treatment and a confirmed diagnosis of BTC, including histological confirmation of malignant disease. Patients with prior treatment for BTC (surgery, radiation, or chemotherapy) were excluded. Likewise, patients with other cancers (except non-melanoma skin cancer and radically treated cancers with no sign of relapse) diagnosed before or within 2 years after diagnosis of BTC were excluded.

A control group consisted of healthy blood donors (n = 180) and patients with benign biliary tract disease (BBTD) (choledocholithiasis, elevated liver enzymes/jaundice, or acute cholangitis) who had an endoscopic retrograde cholangiopancreatography performed (n = 49).

Lastly, a cohort of patients with BTC (n = 8) and controls (n = 394) from a prospective biomarker study (the MICA study) were included. All participants had been referred for a diagnostic work-up due to symptoms, raising suspicion of possible cancer (*e.g.*, abdominal pain, fatigue, weight loss). Participants without a cancer diagnosis after a minimum of 2 years' follow-up were eligible as controls.

None of the control groups included patients with primary sclerosing cholangitis. Thorough descriptions of each study are included in the supplementary materials and methods.

Ethics

Written informed consent was obtained from all participants. The present biomarker study and original clinical studies all complied with the tenets of the Helsinki Declaration (as revised in 2013) and were approved by the institutional review board of the Regional Danish Ethics Committee: H-3-2014-055 (CHOCA), H-3-2010-053 (GI1003), H-2-2014-026 (GI1333), KA-20060113 (BIOPAC), S-20100051 (GOC-BP), S-20080081 (GOX-P), H-7-2014-011 (MICA), H-15017822 (BBTD cohort).

Cohorts

Patients with BTC and controls were divided into a discovery cohort and two validation cohorts according to a prespecified plan (Fig. 1): The discovery cohort included eligible patients from Herlev Hospital (n = 158) and patients from Rigshospitalet with blood samples collected before January 1, 2016 (n = 33). As controls, half of the patients with BBTD (n = 25) and half of the healthy blood donors (n = 90) were used. Furthermore, all non-cancer controls from the MICA study included prior to March 15, 2017 (n = 135) were included in the discovery cohort. To generate protein signatures, the discovery cohort was divided randomly into a detection set (two-thirds of participants) and a replication set (one-third of participants).

Validation cohort 1 consisted of patients with BTC included at Vejle Hospital (n = 99) and patients from Rigshospitalet included after January 1, 2016 (n = 15). The controls in the cohort were the remaining half of patients with BBTD (n = 24), healthy blood donors (n = 90) and the non-cancer patients included in the MICA study between March 15, 2017 and July 1, 2018 (n = 127).

Validation cohort 2 only included BTC (N = 8) and non-cancer controls included after July 1, 2018 in the prospective MICA study (N = 132). All patients had cancer-related symptoms at referral and at time of blood sample collection, and BTC was diagnosed between 1 and 1,178 days (median 12 days) after the sample was collected. The cohort was designed to simulate a real-world clinical setting in which a biomarker could be used to differentiate patients with BTC from those without cancer.

Sample characteristics

All samples were collected prior to initiation of therapy. Serum samples were used in most participants; however, serum samples were not available from patients included at Vejle Hospital; in these patients ethylenediaminetetraacetic acid (EDTA) plasma was used.

The serum samples were prepared by centrifuging blood samples within 3 h after blood was drawn at 2,300 G at 4 °C for 10 min, and serum was then aliquoted in Greiner tubes (Cryo.s™ Freezing Tubes, 2 ml, GR-121280, Greiner Bio-One GmbH, Frickenhausen, Germany). The EDTA plasma samples from Vejle Hospital were collected in 9 ml EDTA tubes and centrifuged within 4 h after blood was drawn at 1,486 G at 21 °C for 10 min and transferred to 15 ml cryo tubes. The samples were subsequently stored at −80 °C.

CA19-9 analyses

Samples from most patients with BTC and all MICA participants were analyzed for CA19-9 prospectively as part of the routine work-up at the same time point as the biobank samples were collected. Samples collected at Rigshospitalet (BIOPAC study) and Herlev Hospital (GI1003, GI1333, CHOCA and MICA studies) were analyzed at the Department of Biochemistry, Herlev Hospital during the same period using the same laboratory equipment

Table 1. Baseline patient characteristics.

	Discovery cohort	Validation cohort 1	Validation cohort 2
**Biliary tract cancer**			
Number of patients	186	113	8
Female	104 (55.9)	72 (63.7)	3 (37.5)
Male	82 (44.1)	41 (36.3)	5 (62.5)
Age (median [IQR])	67 [58, 71]	66 [57, 73]	77 [74, 83]
iCCA	92 (49.5)	38 (33.6)	5 (62.5)
pCCA	21 (11.3)	18 (15.9)	1 (12.5)
dCCA	40 (21.5)	30 (26.5)	0 (0.0)
GBC	33 (17.7)	18 (15.9)	2 (25.0)
Unknown location	0 (0.0)	9 (8.0)	0 (0.0)
Resectable	27 (14.5)	13 (11.5)	1 (12.5)
Locally advanced	76 (40.9)	21 (18.6)	1 (12.5)
Metastatic disease	83 (44.6)	79 (69.9)	6 (75.0)
PS 0	92 (49.5)	43 (38.1)	5 (62.5)
PS 1	78 (41.9)	47 (41.6)	1 (12.5)
PS 2	5 (2.7)	20 (17.7)	1 (12.5)
PS 3	0 (0.0)	0 (0.0)	1 (12.5)
Unknown PS	11 (5.9)	3 (2.7)	0 (0.0)
CA19-9 (median [range])	188 [1, 297,000]	145 [2, 101,263]	29.50 [1, 3,280]
**Healthy blood donors**			
Number of controls	90	90	0
Female	45 (50.0)	40 (44.4)	–
Male	45 (50.0)	50 (55.6)	–
Age (median [IQR])	62 [57, 65]	63 [56, 65]	–
CA19-9 (median [range])	3.00 [1, 159]	2.00 [1, 35]	
**Benign biliary tract disease**			
Number of patients	24	24	0
Female	17 (70.8)	18 (75.0)	–
Male	7 (29.2)	6 (25.0)	–
Age (median [IQR])	47 [36, 60]	46 [38, 62]	–
PS 0	19 (79.2)	20 (83.3)	–
PS 1	3 (12.5)	2 (8.3)	–
PS 2	2 (8.3)	2 (8.3)	–
PS 3	0 (0.0)	0 (0.0)	–
CA19-9 (median [range])	6.00 [1,34]	6.50 [1,36]	
**MICA study: non-cancer controls***			
Number of patients	135	127	132
Female	78 (57.8)	72 (56.7)	80 (60.6)
Male	57 (42.2)	55 (43.3)	52 (39.4)
Age (median [IQR])	60 [50, 70]	65 [54, 72]	68 [57, 73]
PS 0	124 (91.9)	109 (85.8)	100 (75.8)
PS 1	8 (5.9)	18 (14.2)	25 (18.9)
PS 2	2 (1.5)	0 (0.0)	7 (5.3)
PS 3	1 (0.7)	0 (0.0)	0 (0.0)
CA19-9 (median [range])	9 [1, 4440]	9 [1, 315]	1 [1, 723]

CA19-9, carbohydrate antigen 19-9; dCCA, distal cholangiocarcinoma; iCCA, intrahepatic cholangiocarcinoma; pCCA, perihilar cholangiocarcinoma; GBC, gallbladder cancer; PS, performance status.
* Participants referred due to symptoms raising suspicion of possible cancer, but no cancer detected after a minimum of 2 years.

and standard operating procedures. Samples from patients with BTC who did not have available results for CA19-9 (n = 14), all patients with BBTD (n = 49), and healthy participants (n = 180) were analyzed in January 2021 and August 2021. Samples were analyzed at the Department of Clinical Biochemistry, Herlev Hospital using Attelica IM CA19-9 (Siemens Healthcare Diagnostics, Duisburg, Germany), a two-step sandwich type chemiluminescent immunometric assay. The intra-laboratory coefficient of variation using internal controls ranged between 5.7 and 6.2%. Elevated CA19-9 was defined as >37 kU/L.

Protein analyses

A total of 92 proteins were analyzed using the proximity extension assay Immuno-Oncology (I-O) panel from Olink Proteomics, Uppsala, Sweden (www.olink.com). Concentration was measured using an arbitrary unit (normalized protein expression [NPX]) on a Log2 scale. A high NPX value corresponds to a high protein concentration.[30] The proximity extension assay platform has been widely used for biomarker studies, the method has high accuracy and results obtained using this method correlate well with results obtained using other platforms such as multiple reaction monitoring–mass spectrometry and enzyme-linked immunosorbent assays.[30–32] This panel was chosen because BTC is characterized by a high degree of inflammation and an immunosuppressive environment,[6,17,18,22] and the Olink I-O panel has previously been used to generate potential diagnostic protein signatures in pancreatic ductal adenocarcinoma.[26] The analyses were performed blinded at BioXpedia, Aarhus, Denmark according to the manufacturer's instructions. The samples from the healthy blood donors were analyzed using the older version

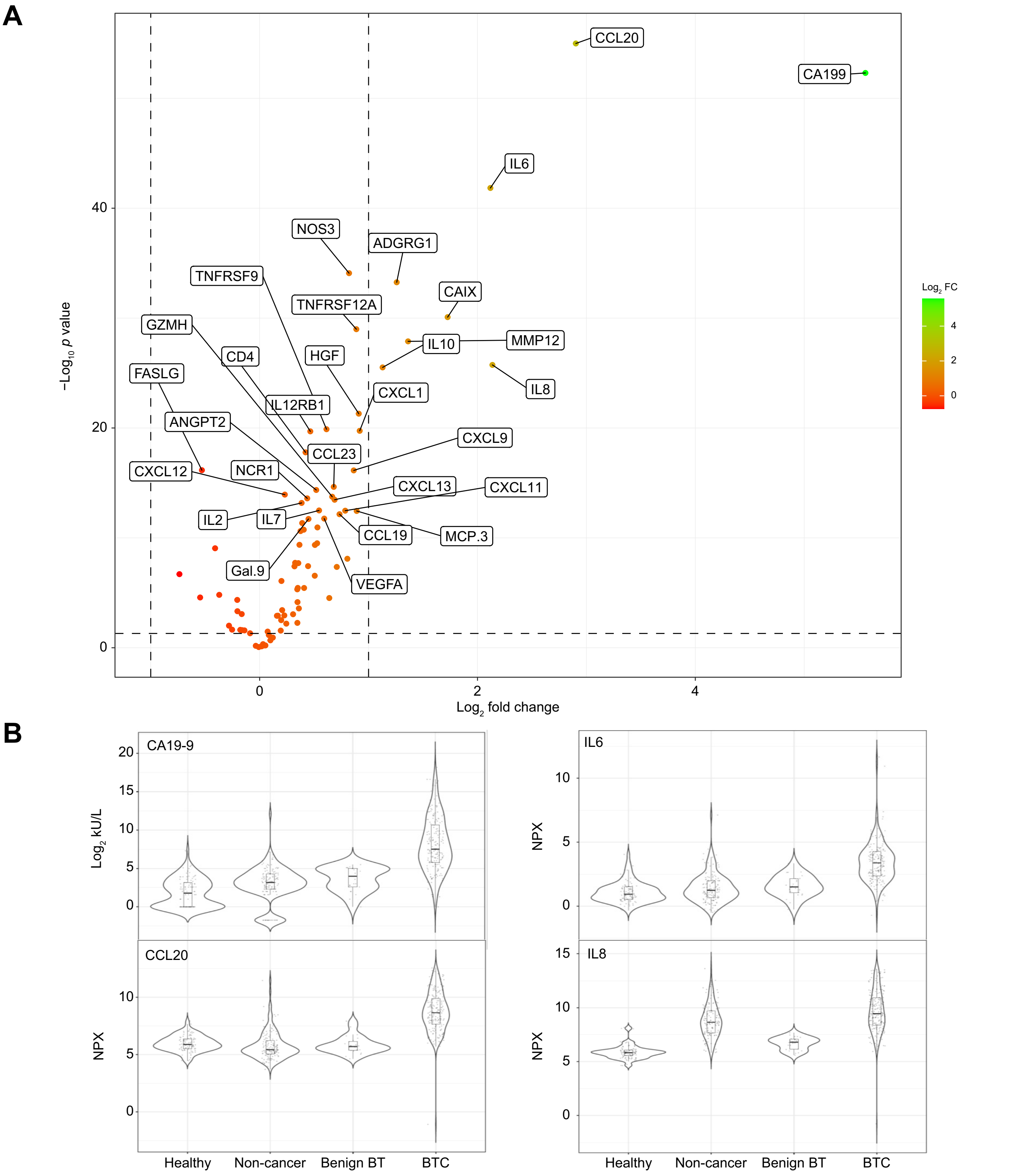

Fig. 2. Protein levels in patients with BTC *vs.* controls in the discovery cohort. (A) Volcano plots showing difference (log2 fold-change) in protein level between patients with BTC and all controls for all 82 proteins. Protein levels compared using a *t* test or Wilcoxon rank sum test where appropriate. The 30 proteins with the lowest *p* value are tagged with their name. Vertical dashed lines indicate a log2 fold-change of 1, horizontal dashed line a *p* value of more than 0.05. (B) Boxplots showing difference in protein level between patients with BTC and the three subgroups of controls for the four proteins with largest overall difference. Benign BT, benign biliary tract disease; BTC, biliary tract cancer, CA19-9, carbohydrate antigen 19-9; CCL20, chemokine (C-C motif) ligand 20; Healthy, healthy blood donors; IL, interleukin; non-cancer, non-cancer controls.

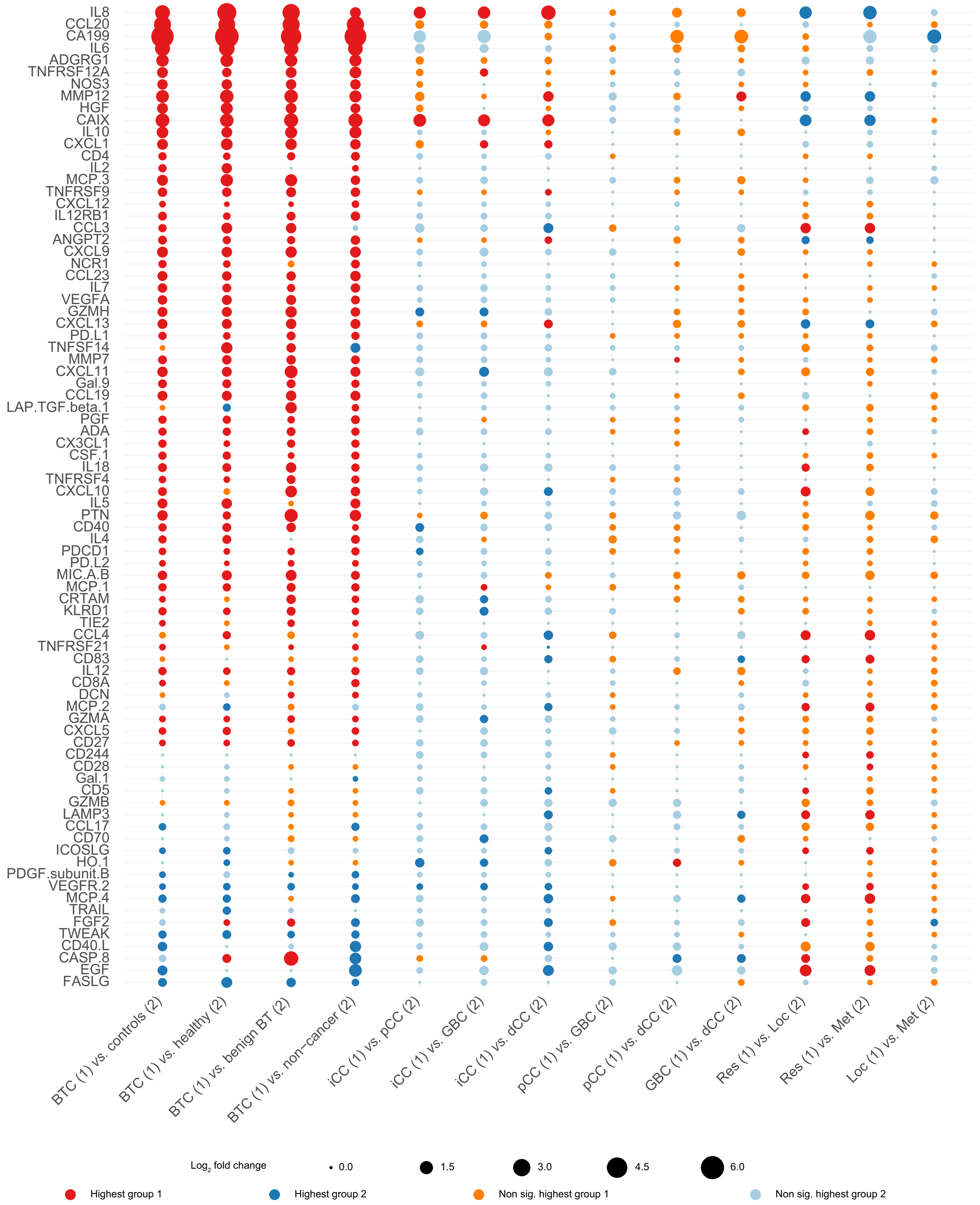

Fig. 3. Difference in circulating protein levels between groups of interest in the discovery cohort. Dot plot showing difference in protein level between patients with BTC and subgroups of controls, and between subgroups of patients with BTC. The size of the dot indicates difference in protein level measured as log2 fold-change. For each dot, the color indicates in which group the protein level is highest and whether differences were significant, tested using *t* test or Wilcoxon rank sum test where appropriate. Significant (adjusted *p* value <0.05) differences are dark red (highest in group 1) or navy blue (highest group 2). Non-significant differences are light red (highest group 1) or light blue (highest group 2). Benign BT, benign biliary tract disease controls; BTC, biliary tract cancer; dCCA, distal cholangiocarcinoma; GBC, gallbladder cancer; Healthy, healthy blood donors; iCCA, intrahepatic cholangiocarcinoma; Loc, locally advanced BTC; Met, metastatic BTC; non-cancer, non-cancer controls; Res, resectable BTC.

of the I-O panel (Proseek® Multiplex Immuno-Oncology, v. 953101; the protein list is available as Table S1). The remaining samples were analyzed using the newer version of the panel (Olink Target 96 Immuno-Oncology, v. 953111/v.953112, Table S2) in three subsequent runs.

Eighty-one proteins were included in the present study. Six proteins were excluded due to changes in assays between I-O panel versions, and five proteins were excluded because more than 90% of values were missing in at least one run. Seven samples were removed due to high internal control deviation or missing data (six samples from the discovery cohort, and one sample from validation study 1). The remaining samples were included in the study (Fig. 1). Detailed description of the protein analysis and quality control is available in the supplementary materials and methods.

Statistical analysis

No studies have previously described the development of multiprotein predictors in patients with BTC, and exact sample size estimation was not possible. Samples were normalized for any plate effects according to the manufacturer's recommendations. To compare serum and plasma samples, plasma results were adjusted using a previously identified serum/plasma ratio.[33]

Protein levels were compared with a *t* test or Wilcoxon rank sum test where appropriate. *p* values were adjusted with the Benjamini-Hochberg method. The log2 fold-change was calculated on a linear scale using the geometric mean of each group.

To generate protein signatures, protein levels, including CA19-9 levels, were scaled to unit variance and centered to have a mean equal to zero. Samples with less than 10% missing values were imputed using the function impute.knn from the R-package impute.[34] The primary set of protein signatures was identified using the 81 proteins + CA19-9. The second set of signatures was generated using CA19-9 + 42 proteins previously identified to have a consistent serum to plasma variation[33]. The signatures were generated using a multi-step strategy based on a similar approach previously employed by our group in a cohort of patients with pancreatic ductal adenocarcinoma.[26] First, using only the detection set of the discovery cohort, a 500-fold bootstrapped Lasso regression (the R-package glmnet[35]) was performed. For each protein, a proportion score was calculated as the number of times each of the 500 logistic Lasso regression models included that protein as a predictor. The proportion scores were used to generate 21 sets of proteins (signatures). Secondly, signatures were fitted on the detection set using Ridge regression (the R-package glmnet[35]). For each signature, a primary model was fitted to discriminate all patients with BTC from all controls, and secondary models fitted to discriminate between subgroups of patients with BTC and controls. The performance was evaluated using the area under the receiver-operating characteristic curves (AUC). Best point (BP) sensitivity, specificity, positive predictive value and negative predictive value were calculated using the Youden's Index[36] and were used as optimal cut-off values. Cut-off values with a sensitivity or specificity above 0.95 were also identified. All signatures were tested with and without age and CA19-9 added as a covariate, and the DeLong test[37] was used to compare the AUCs in the generated models.

For validation cohort 2, the dataset was sent blinded to the statistician, with no information regarding diagnostic group being given. The statistician employed the signatures using the BP (for all BTC *vs.* all controls) identified in the replication cohort as the threshold for case identification. Afterwards, data were unblinded and performance evaluated. A thorough description of signature generation and evaluation is available in the supplementary materials and methods.

Statistical analyses were performed by a trained bioinformatician (EM) following an analysis plan created prior to initiation of analysis using R (R Core Team (2019. R: A language and environment for statistical computing. R Foundation for Statistical Computing, Vienna, Austria). A two-sided *p* value of 0.05 was considered significant.

Results

Patient characteristics

Patient characteristics of the three cohorts are shown in Table 1. Patients with BTC in the discovery cohort more often had iCCA (49.5% *vs.* 33.6%), resectable or locally advanced disease (55.4% *vs.* 30.1%), and performance status 0 or 1 (91.4% *vs.* 79.7%) than those in validation cohort 1. Of the 41 patients with resectable disease, 39 had dCCA, one had GBC (validation cohort 1), and one had iCCA (validation cohort 2). Across all cohorts, patients with BTC had a median age of 67 years *vs.* 63 years for controls, and more often had performance status 1–2 than controls (52.2% *vs.* 15.8%).

Differences in protein level between patients and controls

For the individual proteins, the serum levels were significantly different for CA19-9 and most of the Olink proteins (64 of 81, 79.0%) for BTC *vs.* controls in the discovery cohort. Eight proteins had a log2 fold increase of more than 1: CA19-9, CCL 20, IL-6, IL-8, carbonic anhydrase IX (CAIX), MMP-12, ADGRG1 (adhesion G-protein coupled receptor G1) and IL-10 (Fig. 2). A similar pattern was observed when comparing BTC with subgroups of controls (Fig. 3). Most of the differences between proteins in subgroups of patient with BTC were small; however, IL-8 and CAIX were more than twice as high (log2 fold-change >1) in iCCA than in dCCA, pCCA, or GBC (Fig. 3). Table S3 shows all comparisons made between groups in the discovery cohort. A similar pattern was observed in validation study 1. Notably, CA19-9, CCL20, IL-6, IL-8, CAIX, MMP-12, ADGRG1, and IL-10 were all among the proteins with the highest difference between patients and controls, and again IL-8 and CAIX were notably higher in patients with iCCA than in patients with extrahepatic BTC (Table S4).

Identification of protein signatures in the discovery cohort

We generated 16 signatures (four signatures were duplicates and were not included in further analysis). The signatures included 2 to 82 proteins. All signatures included CA19-9 and CCL20. The list of proteins, proportion scores, and regression coefficients for all signatures are available in Table S5.

Signatures' ability to discriminate BTC from controls in the discovery cohort and validation cohort 1

All signatures performed well in the detection and replication set of the discovery cohort (Fig. 4). In the replication cohort, AUCs ranged from 0.97 to 0.99, BP sensitivity from 0.95 to 0.98, and BP specificity from 0.90 to 0.96. All signatures performed better than CA19-9 (AUC = 0.92, BP sensitivity = 0.85, BP specificity = 0.87), and signatures including ≥4 proteins showed equal performance (Table 2 and Table S6).

The signatures' performances remained high in validation cohort 1 (AUC ≥0.94) (Fig. 4). BP sensitivity ranged from 0.89 to

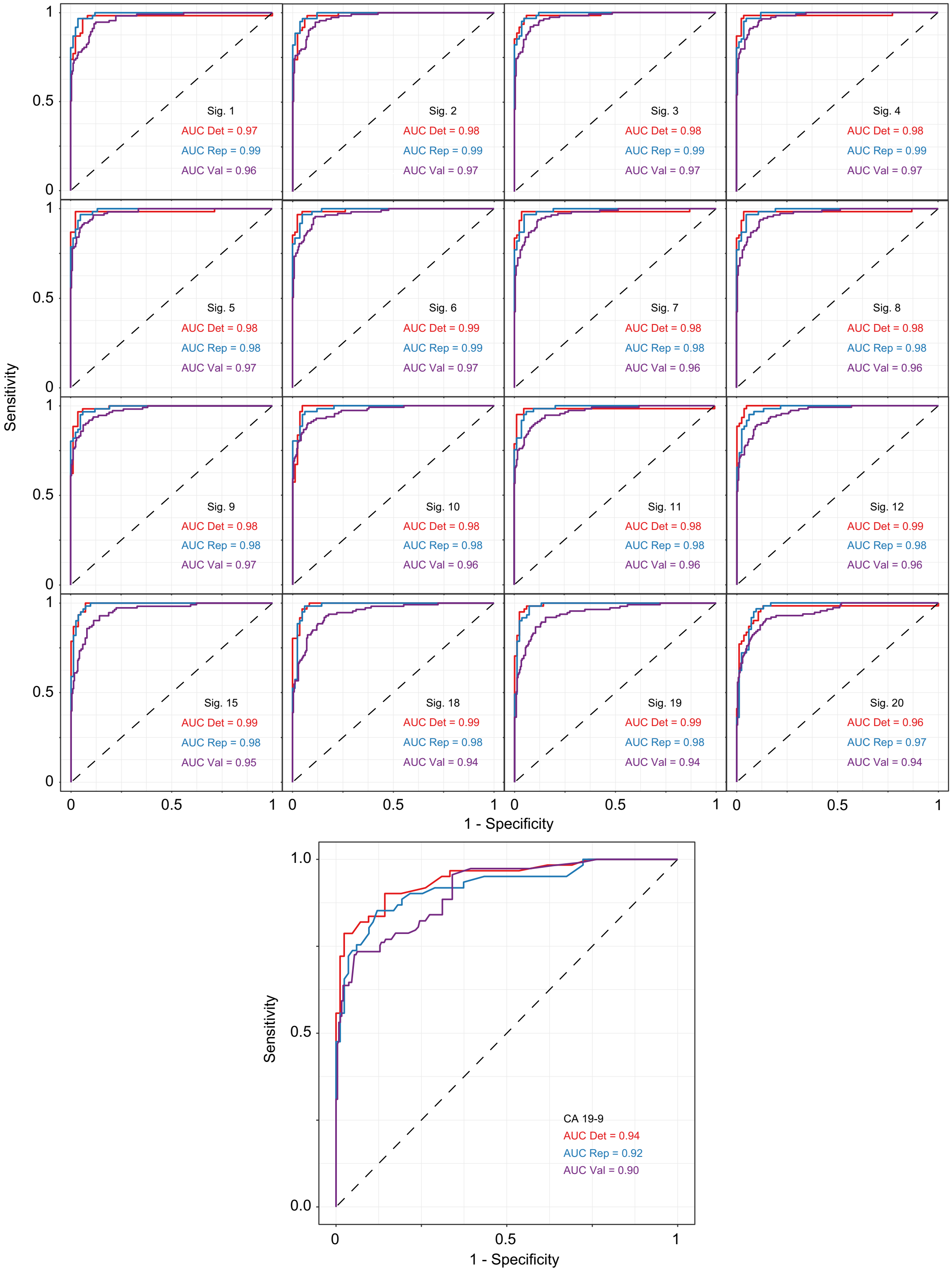

Fig. 4. Signatures' performances for differentiating patients with biliary tract cancer from controls in the discovery cohort and validation cohort 1. Receiver-operating characteristic curve for all signatures and CA19-9. AUC, area under the receiver-operating characteristic curve; CA19-9, carbohydrate antigen 19-9; Det, detection set; Rep, replication set; Sig, signature; and Val, validation cohort 1.

Table 2. Performance of signatures in the discovery cohort and validation cohort 1.

Sig	#	Discovery cohort - detection set				Discovery cohort - replication set				Validation cohort 1			
		AUC	BPse	BPsp	BPt	AUC	BPse	BPsp	BPt	AUC	BPse	BPsp	BPt
1	82	0.97 (0.94–1.00)	0.96 (0.91–1.00)	0.94 (0.88–0.98)	0.42	0.99 (0.98–1.00)	0.96 (0.91–1.00)	0.96 (0.9–1.00)	0.58	0.96 (0.94–0.98)	0.94 (0.89–0.98)	0.87 (0.84–0.92)	0.42
2	61	0.98 (0.97–0.99)	0.98 (0.91–1.00)	0.92 (0.88–0.98)	0.34	0.99 (0.98–1.00)	0.96 (0.91–1.00)	0.95 (0.89–1.00)	0.55	0.97 (0.95–0.98)	0.94 (0.88–0.98)	0.88 (0.85–0.94)	0.45
3	43	0.98 (0.97–1.00)	0.98 (0.91–1.00)	0.94 (0.9–1.00)	0.35	0.99 (0.98–1.00)	0.96 (0.91–1.00)	0.95 (0.89–1.00)	0.5	0.97 (0.95–0.98)	0.92 (0.87–0.98)	0.92 (0.85–0.96)	0.58
4	37	0.98 (0.95–1.00)	0.98 (0.93–1.00)	0.96 (0.92–1.00)	0.4	0.99 (0.98–1.00)	0.96 (0.91–1.00)	0.95 (0.89–1.00)	0.5	0.97 (0.96–0.98)	0.91 (0.88–0.99)	0.93 (0.85–0.96)	0.61
5	32	0.98 (0.96–1.00)	0.98 (0.93–1.00)	0.97 (0.94–1.00)	0.38	0.99 (0.98–1.00)	0.96 (0.91–1.00)	0.95 (0.89–1.00)	0.49	0.97 (0.96–0.99)	0.92 (0.87–0.99)	0.93 (0.86–0.97)	0.59
6	27	0.99 (0.98–1.00)	0.96 (0.93–1.00)	0.97 (0.92–1.00)	0.46	0.99 (0.98–1.00)	0.96 (0.91–1.00)	0.95 (0.89–1.00)	0.55	0.97 (0.95–0.98)	0.95 (0.89–0.99)	0.88 (0.85–0.95)	0.47
7	23	0.98 (0.95–1.00)	0.98 (0.93–1.00)	0.96 (0.91–1.00)	0.42	0.98 (0.97–0.99)	0.96 (0.91–1.00)	0.95 (0.89–1.00)	0.58	0.96 (0.95–0.98)	0.93 (0.85–0.98)	0.88 (0.83–0.95)	0.48
8	19	0.99 (0.97–1.00)	0.98 (0.93–1.00)	0.95 (0.91–1.00)	0.36	0.98 (0.97–0.99)	0.96 (0.91–1.00)	0.95 (0.89–0.98)	0.6	0.96 (0.95–0.98)	0.92 (0.84–0.97)	0.91 (0.85–0.97)	0.58
9	18	0.98 (0.97–1.00)	0.96 (0.93–1.00)	0.96 (0.91–1.00)	0.47	0.98 (0.97–0.99)	0.96 (0.9–1.00)	0.93 (0.89–1.00)	0.56	0.97 (0.95–0.98)	0.89 (0.84–0.97)	0.93 (0.86–0.97)	0.63
10	15	0.98 (0.97–1.00)	1 (0.98–1.00)	0.95 (0.9–0.98)	0.36	0.98 (0.97–0.99)	0.96 (0.91–1.00)	0.93 (0.89–0.98)	0.46	0.96 (0.95–0.98)	0.9 (0.84–0.96)	0.92 (0.87–0.97)	0.59
11	12	0.98 (0.94–1.00)	0.95 (0.91–1.00)	0.98 (0.92–1.00)	0.56	0.98 (0.97–0.99)	0.96 (0.91–1.00)	0.93 (0.89–0.98)	0.47	0.96 (0.94–0.98)	0.9 (0.84–0.97)	0.89 (0.83–0.96)	0.48
12	9	0.99 (0.99–1.00)	1 (0.95–1.00)	0.95 (0.91–1.00)	0.4	0.98 (0.97–0.99)	0.95 (0.88–1.00)	0.93 (0.86–0.98)	0.54	0.96 (0.94–0.97)	0.89 (0.83–0.96)	0.9 (0.82–0.95)	0.47
15	6	0.99 (0.98–1.00)	1 (0.93–1.00)	0.92 (0.88–1.00)	0.36	0.98 (0.97–1.00)	0.98 (0.93–1.00)	0.92 (0.87–1.00)	0.33	0.95 (0.93–0.97)	0.9 (0.84–0.98)	0.88 (0.8–0.94)	0.49
18	5	0.99 (0.98–1.00)	0.96 (0.95–1.00)	0.95 (0.88–0.98)	0.44	0.98 (0.96–1.00)	0.98 (0.93–1.00)	0.93 (0.89–0.98)	0.42	0.94 (0.92–0.96)	0.92 (0.82–0.97)	0.83 (0.79–0.94)	0.4
19	4	0.99 (0.98–1.00)	0.95 (0.91–1.00)	0.97 (0.91–1.00)	0.49	0.98 (0.97–1.00)	0.98 (0.91–1.00)	0.92 (0.87–1.00)	0.41	0.94 (0.91–0.96)	0.92 (0.81–0.95)	0.84 (0.81–0.93)	0.42
20	2	0.96 (0.92–0.99)	0.98 (0.86–1.00)	0.86 (0.82–0.97)	0.29	0.97 (0.95–0.99)	0.96 (0.91–1.00)	0.9 (0.83–0.97)	0.4	0.94 (0.91–0.96)	0.91 (0.8–0.95)	0.85 (0.81–0.95)	0.4
CA 19-9		0.94 (0.9–0.98)	0.78 (0.73–0.96)	0.97 (0.82–1.00)	0.48	0.92 (0.87–0.96)	0.85 (0.72–0.95)	0.87 (0.79–0.98)	0.41	0.9 (0.87–0.94)	0.73 (0.66–0.97)	0.93 (0.66–0.97)	0.57

All values are presented with bootstrapped 95% CIs in parentheses.
AUC, area under receiver-operating characteristic curve; BPse, best point sensitivity; BPsp, best point specificity; BPt, best point threshold; CA19-9, carbohydrate antigen 19-9; Sig, signature.
[#] Number of proteins.

0.94, and BP specificity from 0.83 to 0.93. All signatures achieved a higher AUC than CA19-9 alone (AUC = 0.90), and signatures including ≥9 proteins all had AUC ≥0.96 (Table 2 and Table S6). A secondary set of 17 signatures including CA19-9 and 1 to 42 proteins with stable serum/plasma variation showed equal performance, with AUCs above 0.94 to 0.97 (Table S7).

Excluding CA19-9 from the signatures decreased their performances, especially in validation cohort 1, where all signatures had a significantly lower AUC after excluding CA19-9 (DeLong test, p ≥0.001). After removing CA19-9, the signatures' AUC remained above 0.95 for signatures 1–10 (including 81–14 proteins), and the lowest AUC was 0.85 for signature 20 (CCL20 alone) in validation cohort 1 (Table S8). Adding age as a variable to the signatures did not significantly improve the AUC of the models (DeLongs test, p >0.05) (Data not shown).

Signature's ability to discriminate between subgroups

The signatures were also tested for their ability to identify subgroups of patients with BTC divided according to location (iCCA, dCCA, pCCA, GBC) and stage (resectable, locally advanced, and metastatic disease). For most comparisons of controls *vs.* subgroups of BTC, signatures performed better than CA19-9 alone, and signatures that included ≥4 proteins showed similar performance in both the discovery cohort and validation cohort 1 (Table S9). Notably, signatures discriminated well between early stage BTC and controls in both the replication cohort (AUC 0.96–0.98) and validation cohort 1 (AUC 0.92–0.98), and all achieved a higher AUC than CA19-9 alone.

Likewise, the ability of the signatures to identify patients with BTC from subgroups of controls was tested. Signatures had a high AUC in all cohorts when comparing BTC *vs.* healthy (AUC ≥0.96), BTC *vs.* BBTD (AUC ≥0.94), and BTC *vs.* non-cancer controls from the MICA study (AUC ≥0.93). For comparisons between BTC and non-cancer controls, signatures including ≥12 proteins performed best (AUC ≥0.97) (Table S9).

Blinded prediction in validation cohort 2

Lastly, signatures' performances were evaluated in a real-world setting in validation cohort 2. All participants in this cohort had been referred to the hospital due to cancer-related symptoms (list of symptoms available in Table S10). Using thresholds defined in the replication cohort, 15 of 16 signatures were able to identify all patients with BTC, giving a sensitivity of 100%. Interestingly, one patient with iCCA and one patient with GBC were diagnosed more than a year after the blood sample was collected (17 months and 38 months, respectively). The specificity for the signatures was 71.2% for the best performing signature (signature 1), and in general the performance decreased the fewer proteins were included. CA19-9 had a sensitivity of 87.5% and specificity of 43.5%. The sensitivity, specificity, negative predictive value and positive predictive value are reported in Table 3.

Unblinded test in validation cohort 2

After unblinding, the overall ability of the signatures was assessed using receiver-operating characteristic curves, and the optimal threshold for validation cohort 2 was identified (Fig. 5). The AUC was 0.94 for the best performing signature (signature 6: 27 proteins), with a sensitivity of 100% and specificity of 84%. However, eight of the other signatures were able to achieve a specificity of 82–84% while keeping a sensitivity of 100%. All eight signatures included ≥15 proteins. For comparison, CA19-9

Table 3. Performance of the signatures in validation cohort 2.

Sig	#	Blinded prediction			Unblinded prediction			
		Sensitivity	Specificity	Threshold	AUC	BPse	BPsp	BPt
1	82	1.00	0.71	0.58	0.92 (0.86-0.97)	1.00 (1.00-1.00)	0.82 (0.76-0.94)	0.90
2	61	1.00	0.68	0.55	0.93 (0.88-0.98)	1.00 (1.00-1.00)	0.85 (0.79-0.95)	0.92
3	43	1.00	0.64	0.5	0.93 (0.88-0.98)	1.00 (0.87-1.00)	0.82 (0.76-0.96)	0.88
4	37	1.00	0.61	0.5	0.93 (0.87-0.98)	1.00 (0.87-1.00)	0.80 (0.75-0.95)	0.85
5	32	1.00	0.62	0.49	0.92 (0.87-0.98)	1.00 (0.87-1.00)	0.79 (0.74-0.94)	0.82
6	27	1.00	0.65	0.55	0.94 (0.89-0.98)	1.00 (1.00-1.00)	0.84 (0.79-0.96)	0.88
7	23	1.00	0.69	0.58	0.93 (0.88-0.98)	1.00 (1.00-1.00)	0.83 (0.78-0.94)	0.89
8	19	1.00	0.69	0.6	0.91 (0.86-0.97)	1.00 (1.00-1.00)	0.83 (0.77-0.91)	0.89
9	18	1.00	0.68	0.56	0.91 (0.85-0.96)	1.00 (1.00-1.00)	0.84 (0.78-0.91)	0.89
10	15	1.00	0.64	0.46	0.91 (0.85-0.96)	1.00 (1.00-1.00)	0.84 (0.78-0.91)	0.87
11	12	1.00	0.65	0.47	0.87 (0.80-0.95)	1.00 (0.87-1.00)	0.70 (0.65-0.89)	0.68
12	9	1.00	0.67	0.54	0.87 (0.78-0.95)	1.00 (1.00-1.00)	0.74 (0.67-0.90)	0.69
15	6	1.00	0.48	0.33	0.84 (0.75-0.94)	1.00 (0.87-1.00)	0.64 (0.58-0.89)	0.51
18	5	1.00	0.58	0.42	0.85 (0.76-0.94)	1.00 (0.87-1.00)	0.67 (0.61-0.85)	0.52
19	4	0.88	0.53	0.41	0.85 (0.74-0.96)	0.87 (0.75-1.00)	0.82 (0.50-0.90)	0.74
20	2	1.00	0.52	0.4	0.84 (0.72-0.95)	1.00 (0.75-1.00)	0.61 (0.54-0.94)	0.49
CA19-9		0.88	0.44	0.41	0.73 (0.50-0.95)	0.75 (0.37-1.00)	0.68 (0.45-1.00)	0.59

All values are presented with bootstrapped 95% CIs in parentheses.
AUC, area under receiver-operating characteristic curve; BPse, best point sensitivity; BPsp, best point specificity; BPt, best point threshold; CA19-9, carbohydrate antigen 19-9; Sig., signature.
[#] Number of proteins.

had an AUC of 0.73, sensitivity of 75%, and specificity of 68% (Table 3).

Discussion

Our study demonstrated that it is possible to generate multi-protein blood signatures that can differentiate patients with BTC from non-cancer controls with high sensitivity and reasonable specificity. In general, combining several proteins improved performance compared to single or dual markers, but adding more than 15 proteins to the signatures did not improve the performance significantly in any of our cohorts. Importantly, the results were reproducible in two validation cohorts, including a cohort representing a real-world setting of patients suspected of having cancer.

An approach like ours, where diagnostic blood-based signatures were created using protein panels covering multiple inflammation-related proteins (>10), has only been tested in patients with GBC. Koshiol *et al.* measured the levels of several inflammation-related protein markers in 150 patients with GBC and compared them to levels in patients with gallstones. They created a protein signature based on four proteins (IL-6, IL-16, CCL20, and soluble tumor necrosis factor receptor-1). The score achieved a specificity of 66.6% at a sensitivity of 90% for detecting early GBC [21]; however, no validation study was performed. Notably, two of the same proteins (CCL20 and IL-6) were

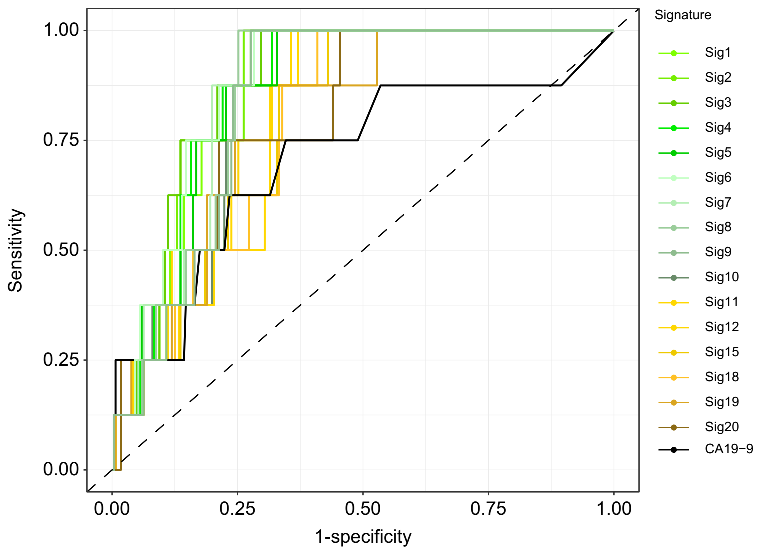

Fig. 5. Signatures' performances for differentiating patients with biliary tract cancer from controls in validation cohort 2. Receiver-operating characteristic curve for all signatures and CA19-9. CA19-9, carbohydrate antigen 19-9; Sig, signature.

included in all our signatures.[21] Other studies investigating the use of multiprotein signatures as novel biomarkers are limited by a small number of patients with BTC (n <70) and a lack of validation in independent cohorts.[38–42]

Other blood-based multiplex biomarkers have shown promising performance in detecting patients with BTC. Hu *et al.* performed a study using 359 patients with iCCA and 642 controls. They identified a three-marker model that included miR-21, miR-122, and CA19-9 that achieved an AUC of 0.866 in their validation cohort.[43] Tumor-associated microparticles and serum metabolites have been used for multi-marker signatures with potential diagnostic potential.[44,45] Although the studies are not directly compatible with ours, the protein signatures we identified showed equal or better performance than previous multiplex biomarkers.

We previously found a potential diagnostic signature for patients with pancreatic ductal adenocarcinoma using the Olink I-O panel. The signature included several of the proteins included in our study, including CA19-9, CCL20, caspase-8, and Fas ligand. However, other proteins such as TWEAK (tumor necrosis factor ligand superfamily member 12), NCR1 (natural cytotoxicity triggering receptor) and IL-6, which were widely used in our signatures, were not included in the pancreatic ductal adenocarcinoma signature.[26]

The Olink I-O panel targets proteins related to cancer and the immune system and several of the proteins we studied have previously been found elevated in BTC.[21,23] This is in line with our findings, as most of the proteins were elevated in patients with BTC compared with controls. Besides CA19-9, the most prominent was the chemokine CCL20, whose levels in plasma were more than fourfold higher in patients with BTC than in controls in both the discovery and validation cohorts. Both tumor and stromal cells produce CCL20, which, together with its receptor CCR6 (C-C chemokine receptor 6), is involved in leucocyte migration and inflammation. It is associated with treatment resistance and survival, suggesting that the CCL20-CCR6 axis may be a potential treatment target.[20,46] Interestingly, CCL20 is expressed more commonly in CCA cells than in normal adjacent tissue.[47] Two studies found elevated CCL20 in patients with GBC compared to patients with gallstones,[21,23] but our study is the first to show and validate an increased plasma level and potential diagnostic use of CCL20 in patients with CCA. We also found that IL-6 and IL-8 were markedly elevated in patients with BTC compared to controls. Both interleukins have previously been associated with BTC outcome, and IL-6 has been suggested as a treatment target.[21,22,48,49] Several other proteins found to be significantly elevated in patients with BTC have also been found to be elevated in patients with GBC (CCL4, CCL19, CXCL9, CXCL10, CXCL11, CXCL13, IL-10).[21]

The different subtypes of BTC (iCCA, pCCA, dCCA, and GBC) are often characterized as distinct diseases with different molecular alterations, but they also share several similarities, including a high degree of local and systemic inflammation.[6,17,18,21–23] Notably, the protein signatures performed well in identifying patients with BTC regardless of location, and only minor differences in plasma level were observed between patients with BTC with regard to the individual proteins. The most notable difference was a higher level of CAIX and IL-8 in patients with iCCA than in extrahepatic subtypes. The signatures were also able to identify patients with early stage resectable disease from controls in both the discovery cohort and validation cohort 1. Most patients with resectable disease had dCCA, limiting our ability to evaluate the performance at identifying early stage iCCA, pCCA, or GBC. However, results from validation cohort 2 indicate that our signatures would be able to identify these patients. Here, the signature was able to identify one patient with stage I iCCA and two patients diagnosed with advanced iCCA and GBC more than a year after samples were collected.

The optimal threshold of the signatures for identification of patients with BTC from controls was similar in the discovery cohort and validation cohort 1. Using the threshold identified in validation cohort 2, most signatures achieved a sensitivity of 100%, but the optimal threshold after unblinding was identified as being higher. The explanation could be that validation cohort 2 was imbalanced compared to the other studies, included very few patients with BTC, and had only controls from the MICA study. The optimal threshold was therefore not fully established in this study and should probably be identified for each population of interest.

This study has some limitations. First, the analyzed blood samples from all controls in validation cohort 1 were serum samples, whereas EDTA plasma samples were used from 99 of the 114 patients with BTC, introducing a potential bias. However, the effect of this on the conclusion was probably minor. Similar results were obtained in validation cohort 1 when only proteins with a stable serum/plasma ratio were analyzed. Likewise, signatures performed well in validation cohort 1 when performance was tested in the 15 early stage patients using only serum samples. Second, the control cohort of patients with BBTD had low CA19-9 levels, and the expression of most proteins was more similar to that observed in the two other control groups than that observed in patients with BTC. The reason might be that patients were included doing follow-up at a time when their biliary disease could be in remission. Third, we did not include controls with known risk factors, such as primary sclerosing cholangitis. Therefore, we do not know whether our signatures can distinguish acute BBTD from BTC or identify patients with BTC among high-risk patients with, for example, primary sclerosing cholangitis. Future studies should explore this. Fourth, CA19-9 was not measured using the same laboratory equipment for all patients and controls, introducing a potential bias. However, we do not believe this affected the overall conclusions substantially since most samples were analyzed in the same laboratory (Herlev Hospital), and the signatures achieved a high AUC even after removing CA19-9. Lastly, protein levels of the I-O proteins were only measured using the Olink panel. The reproducibility of our results using other protein detection methods is not known. Furthermore, the Olink panel measures protein level as relative abundance and consequently, thresholds and protein levels observed in this study are not directly transferable to other studies.

The protein signatures were able to identify patients with BTC in individuals referred for a diagnostic work-up due to suspicion of cancer in all three cohorts. In both validation cohorts 1 and 2, signatures including ≥15 proteins showed the best performance. Given the high sensitivity of the models in this setting, protein biomarkers like these could be used to rule out cancer in such a setting or select patients for intensified surveillance programs. The protein signatures might also have a use in patients with a radiologically proven liver tumor, but where a usable biopsy is

not possible due to the location of the tumor. Here, such a biomarker could support a diagnosis of BTC and decisions on a treatment strategy that would lead to earlier treatment initiation and a better outcome.

In conclusion, our study identified new potential diagnostic blood-based protein signatures that may help identify patients with BTC from patients without cancer. Although the protein signatures we used need further independent validation, they showed promising performance. The study also validated the use of multiprotein diagnostic cancer biomarkers based on proximity extension assay technology. The next step in the development of a clinically useful biomarker is to investigate how well the signatures can distinguish patients with BTC from patients with other cancers.

Abbreviations

AUC, area under receiver-operating characteristic curve; BBTD, benign biliary tract disease; BP, best point; BTC, biliary tract cancer; CA19-9, carbohydrate antigen 19-9; CAIX, carbonic anhydrase IX; CASP8, caspase 8; CCA, cholangiocarcinoma; CCL, chemokine (C-C motif) ligand; CXCR, C-X-C motif chemokine; dCCA, distal cholangiocarcinoma; EDTA, ethylenediaminetetraacetic acid; GBC, gall bladder cancer; iCCA, intrahepatic cholangiocarcinoma; IL, interleukin; I-O, immuno-oncology; MMP-, matrix metalloproteinase-; NPX, normalized protein expression; pCCA, perihilar cholangiocarcinoma; TME, tumor microenvironment.

Financial support

The Danish Cancer Society (grant number R218-A13148) provided salary for TDC. Funding for protein analyses and statistical analyses was provided by Beckett-Fonden, Fonden til fremme af klinisk cancerforskning, The A.P. Moller Foundation, and Tømrermester Holms Mindelegat. The funding sources were not involved in the study design; the collection, analysis, interpretation of the data; or writing of the report.

Conflict of interest

The authors declare no conflicts of interest that pertain to this work.

Please refer to the accompanying ICMJE disclosure forms for further details.

Authors' contributions

All authors have accepted responsibility for the entire content of this manuscript and approved its submission. TDC, OL, IMC, DN, and JSJ formulated the initial idea for the project and together with EM drafted the analysis plan. OL, CLF, KRN, LHJ, BL, JSJ and CPH were responsible for the original trials and inclusion of patients. TDC, CLF, KRN, LHJ, BL, and JSJ performed data curation. TDC and JSJ were responsible for identifying suitable samples. EM performed the bioinformatic interpretation and main statistical analysis in collaboration with TDC. TDC performed the initial draft of the manuscript. All authors reviewed and contributed to the final manuscript. All authors read and approved the final manuscript.

Data availability statement

Data are available upon reasonable request. Data cannot be uploaded online due to regulations by the Danish Data Protection Agency.

Acknowledgments

We thank our colleagues at all the sites for their assistance with inclusion of patients. Likewise, we thank The Danish Cancer Society, Beckett-Fonden, Fonden til fremme af klinisk cancerforskning, The A.P. Moller Foundation, and Tømrermester Holms Mindelegat for their financial contributions to our project. Many thanks to the biomedical laboratory scientists Charlotte Falk, Vibeke H. Holm, Marta Monika Asadi Sohi and Astrid Z. Johansen for their assistance with the blood sample handling. We thank Edwin Stanton Spencer for English language editing.

Supplementary data

Supplementary data to this article can be found online at https://doi.org/10.1016/j.jhepr.2022.100648.

References

Author names in bold designate shared co-first authorship

[1] Van Dyke AL, Shiels MS, Jones GS, Pfeiffer RM, Petrick JL, Beebe-Dimmer JL, et al. Biliary tract cancer incidence and trends in the United States by demographic group, 1999-2013. Cancer 2019;125:1489–1498.

[2] Ferlay J, Soerjomataram I, Dikshit R, Eser S, Mathers C, Rebelo M, et al. Cancer incidence and mortality worldwide: sources, methods and major patterns in GLOBOCAN 2012. Int J Cancer 2015;136:E359–E386.

[3] Banales JM, Cardinale V, Carpino G, Marzioni M, Andersen JB, Invernizzi P, et al. Cholangiocarcinoma: current knowledge and future perspectives consensus statement from the European Network for the Study of Cholangiocarcinoma (ENS-CCA). Nat Rev Gastroenterol Hepatol 2016;13:261–280.

[4] Ferlay J, Colombet M, Soerjomataram I, Dyba T, Randi G, Bettio M, et al. Cancer incidence and mortality patterns in Europe: estimates for 40 countries and 25 major cancers in 2018. Eur J Cancer 2018;103:356–387.

[5] Saha SK, Zhu AX, Fuchs CS, Brooks GA. Forty-year trends in cholangiocarcinoma incidence in the U.S.: intrahepatic disease on the rise. Oncologist 2016;21:594–599.

[6] Valle JW, Kelley RK, Nervi B, Oh D-Y, Zhu AX. Biliary tract cancer. Lancet 2021;397:428–444.

[7] Pinter M, Hucke F, Zielonke N, Waldhör T, Trauner M, Peck-Radosavljevic M, et al. Incidence and mortality trends for biliary tract cancers in Austria. Liver Int 2014;34:1102–1108.

[8] Kim B-W, Oh C-M, Choi HY, Park J-W, Cho H, Ki M. Incidence and overall survival of biliary Tract cancers in South Korea from 2006 to 2015: using the national health information database. Gut Liver 2019;13:104–113.

[9] Flemming JA, Zhang-Salomons J, Nanji S, Booth CM. Increased incidence but improved median overall survival for biliary tract cancers diagnosed in Ontario from 1994 through 2012: a population-based study. Cancer 2016;122:2534–2543.

[10] Rawla P, Sunkara T, Thandra KC, Barsouk A. Epidemiology of gallbladder cancer. Clin Exp Hepatol 2019;5:93–102.

[11] Razumilava N, Gores GJ. Cholangiocarcinoma. Lancet 2014;383:2168–2179.

[12] Blechacz B. Cholangiocarcinoma: current knowledge and new developments. Gut Liver 2017;11:13–26.

[13] Cillo U, Fondevila C, Donadon M, Gringeri E, Mocchegiani F, Schlitt HJ, et al. Surgery for cholangiocarcinoma. Liver Int 2019;39(Suppl 1):143–155.

[14] Liang B, Zhong L, He Q, Wang S, Pan Z, Wang T, et al. Diagnostic accuracy of serum CA19-9 in patients with cholangiocarcinoma: a systematic review and meta-analysis. Med Sci Monit 2015;21:3555–3563.

[15] Macias RIR, Kornek M, Rodrigues PM, Paiva NA, Castro RE, Urban S, et al. Diagnostic and prognostic biomarkers in cholangiocarcinoma. Liver Int 2019;39:108–122.

[16] Sia D, Hoshida Y, Villanueva A, Roayaie S, Ferrer J, Tabak B, et al. Integrative molecular analysis of intrahepatic cholangiocarcinoma reveals 2 classes that have different outcomes. Gastroenterology 2013;144:829–840.

[17] Fabris L, Perugorria MJ, Mertens J, Björkström NK, Cramer T, Lleo A, et al. The tumour microenvironment and immune milieu of cholangiocarcinoma. Liver Int 2019;39:63–78.

[18] Høgdall D, Lewinska M, Andersen JB. Desmoplastic tumor microenvironment and immunotherapy in cholangiocarcinoma. Trends Cancer 2018;4:239–255.

[19] Affo S, Yu L-X, Schwabe RF. The role of cancer-associated fibroblasts and fibrosis in liver cancer. Annu Rev Pathol 2017;12:153–186.

[20] Kadomoto S, Izumi K, Mizokami A. The CCL20-CCR6 axis in cancer progression. Int J Mol Sci 2020;21:5186.
[21] Koshiol J, Gao Y-T, Corbel A, Kemp TJ, Shen M-C, Hildesheim A, et al. Circulating inflammatory proteins and gallbladder cancer: potential for risk stratification to improve prioritization for cholecystectomy in high-risk regions. Cancer Epidemiol 2018;54:25–30.
[22] Høgdall D, O'Rourke CJ, Dehlendorff C, Larsen OF, Jensen LH, Johansen AZ, et al. Serum IL6 as a prognostic biomarker and IL6R as a therapeutic target in biliary tract cancers. Clin Cancer Res : official J Am Assoc Cancer Res 2020;26:5655–5667.
[23] Koshiol J, Castro F, Kemp TJ, Gao YT, Roa JC, Wang B, et al. Association of inflammatory and other immune markers with gallbladder cancer: results from two independent case-control studies. Cytokine 2016;83:217–225.
[24] Leelawat K, Sakchinabut S, Narong S, Wannaprasert J. Detection of serum MMP-7 and MMP-9 in cholangiocarcinoma patients: evaluation of diagnostic accuracy. BMC Gastroenterol 2009;9. 30-30.
[25] Arbelaiz A, Azkargorta M, Krawczyk M, Santos-Laso A, Lapitz A, Perugorria MJ, et al. Serum extracellular vesicles contain protein biomarkers for primary sclerosing cholangitis and cholangiocarcinoma. Hepatology 2017;66:1125–1143.
[26] Lindgaard SC, Sztupinszki Z, Maag E, Chen IM, Johansen AZ, Jensen BV, et al. Circulating protein biomarkers for use in pancreatic ductal adenocarcinoma identification. Clin Cancer Res Official J Am Assoc Cancer Res 2021;27:2592–2603.
[27] Enroth S, Berggrund M, Lycke M, Broberg J, Lundberg M, Assarsson E, et al. High throughput proteomics identifies a high-accuracy 11 plasma protein biomarker signature for ovarian cancer. Commun Biol 2019;2:221.
[28] Skubitz APN, Boylan KLM, Geschwind K, Cao Q, Starr TK, Geller MA, et al. Simultaneous measurement of 92 serum protein biomarkers for the development of a multiprotein classifier for ovarian cancer detection. Cancer Prev Res 2019;12:171.
[29] Collins GS, Reitsma JB, Altman DG, Moons KGM. Transparent reporting of a multivariable prediction model for individual prognosis or diagnosis (TRIPOD): the TRIPOD Statement. BMC Med 2015;13. 1-1.
[30] **Assarsson E**, **Lundberg M**, Holmquist G, Björkesten J, Thorsen SB, Ekman D, et al. Homogenous 96-plex PEA immunoassay exhibiting high sensitivity, specificity, and excellent scalability. PLoS One 2014;9:e95192.
[31] Bhardwaj M, Terzer T, Schrotz-King P, Brenner H. Comparison of proteomic technologies for blood-based detection of colorectal cancer. Int J Mol Sci 2021;22:1189.
[32] Lindgaard SC, Maag E, Sztupinszki Z, Chen IM, Johansen AZ, Jensen BV, et al. Circulating protein biomarkers for prognostic use in patients with advanced pancreatic ductal adenocarcinoma undergoing chemotherapy. Cancers (Basel) 2022;14.
[33] Christensen TD, Maag E, Madsen K, Lindgaard SC, Nielsen D, Johansen JS. Determination of temporal reproducibility and variability of cancer biomarkers in serum and EDTA plasma samples using a proximity extension assay. Clin Proteomics 2022;19:39.
[34] Hastie T, Tibshirani R, Narasimhan B, Chu G. impute: imputation for microarray data. 2019. R package version 1.58.0.
[35] Friedman J, Hastie T, Tibshirani R. Regularization paths for generalized linear models via coordinate descent. J Stat Softw 2010;33:1–22.
[36] Ruopp MD, Perkins NJ, Whitcomb BW, Schisterman EF. Youden Index and optimal cut-point estimated from observations affected by a lower limit of detection. Biom J 2008;50:419–430.
[37] DeLong ER, DeLong DM, Clarke-Pearson DL. Comparing the areas under two or more correlated receiver operating characteristic curves: a nonparametric approach. Biometrics 1988;44:837–845.
[38] Kimawaha P, Jusakul A, Junsawang P, Thanan R, Titapun A, Khuntikeo N, et al. Establishment of a potential serum biomarker panel for the diagnosis and prognosis of cholangiocarcinoma using decision tree algorithms. Diagnostics (Basel) 2021:11.
[39] Li Y, Li DJ, Chen J, Liu W, Li JW, Jiang P, et al. Application of joint detection of AFP, CA19-9, CA125 and CEA in identification and diagnosis of cholangiocarcinoma. Asian Pac J Cancer Prev 2015;16:3451–3455.
[40] Lumachi F, Lo Re G, Tozzoli R, D'Aurizio F, Facomer F, Chiara GB, et al. Measurement of serum carcinoembryonic antigen, carbohydrate antigen 19-9, cytokeratin-19 fragment and matrix metalloproteinase-7 for detecting cholangiocarcinoma: a preliminary case-control study. Anticancer Res 2014;34:6663–6667.
[41] Mou Y, Xing R, Liu C. Diagnosis of gallbladder cancer using matrix-assisted laser desorption/ionization time-of-flight profiling. Am J Med Sci 2012;343:119–123.
[42] Cuenco J, Wehnert N, Blyuss O, Kazarian A, Whitwell HJ, Menon U, et al. Identification of a serum biomarker panel for the differential diagnosis of cholangiocarcinoma and primary sclerosing cholangitis. Oncotarget 2018;9:17430–17442.
[43] Hu J, Wang YN, Song DJ, Tan JP, Cao Y, Fan J, et al. A high-accuracy model based on plasma miRNAs diagnoses intrahepatic cholangiocarcinoma: a single center with 1001 samples. Diagnostics (Basel) 2021:11.
[44] Liang Q, Liu H, Zhang T, Jiang Y, Xing H, Zhang H. Serum metabolomics uncovering specific metabolite signatures of intra- and extrahepatic cholangiocarcinoma. Mol Biosyst 2016;12:334–340.
[45] Julich-Haertel H, Urban SK, Krawczyk M, Willms A, Jankowski K, Patkowski W, et al. Cancer-associated circulating large extracellular vesicles in cholangiocarcinoma and hepatocellular carcinoma. J Hepatol 2017;67:282–292.
[46] Chen KJ, Lin SZ, Zhou L, Xie HY, Zhou WH, Taki-Eldin A, et al. Selective recruitment of regulatory T cell through CCR6-CCL20 in hepatocellular carcinoma fosters tumor progression and predicts poor prognosis. PLoS One 2011;6:e24671.
[47] Win Maung HM, Chan-On W, Kunkeaw N, Khaenam P. Common transcriptional programs and the role of chemokine (C-C motif) ligand 20 (CCL20) in cell migration of cholangiocarcinoma. EXCLI J 2020;19:154–166.
[48] Sun Q, Li F, Sun F, Niu J. Interleukin-8 is a prognostic indicator in human hilar cholangiocarcinoma. Int J Clin Exp Pathol 2015;8:8376–8384.
[49] Nguyen MLT, Bui KC, Scholta T, Xing J, Bhuria V, Sipos B, et al. Targeting interleukin 6 signaling by monoclonal antibody siltuximab on cholangiocarcinoma. J Gastroenterol Hepatol 2021;36:1334–1345.

Journal of Hepatology, Volume *5*

Supplemental information

Development and validation of circulating protein signatures as diagnostic biomarkers for biliary tract cancer

Troels D. Christensen, Emil Maag, Ole Larsen, Claus L. Feltoft, Kaspar René Nielsen, Lars Henrik Jensen, Bonna Leerhøy, Carsten P. Hansen, Inna M. Chen, Dorte L. Nielsen, and Julia S. Johansen

Development and validation of circulating protein signatures as diagnostic biomarkers for biliary tract cancer

Troels D. Christensen, Emil Maag, Ole Larsen, Claus L. Feltoft, Kaspar René Nielsen, Lars Henrik Jensen, Bonna Leerhøy, Carsten P. Hansen, Inna M. Chen, Dorte L. Nielsen, Julia S. Johansen

Table of contents

Supplementary materials and methods...2
Supplementary references.. 8
Supplementary tables..separate excel file

Supplementary materials and methods

Patients

Biliary tract cancer patients from Herlev Hospital, Herlev, Denmark

Patients from Herlev hospital had been included in one of the following studies:

CHOCA study: An ongoing prospective observational open-cohort biomarker study of patients with biliary tract cancer (BTC) treated at the Department of Oncology, Herlev Hospital (Clinical.trials.gov.ID: NCT05184400, Regional Ethics Committee approval number: H-3-2014-055, the Danish Data Protection Agency approval number: BBH-2014-097, I-suite nr. 03260, P-2020-797). All patients referred for treatment for BTC were eligible, and patients were included at initiation of adjuvant, neoadjuvant, or palliative treatment (first-line and later-line treatment). They were treated with different types of chemotherapy or protocolized treatment with targeted monoclonal antibodies, immunotherapy, and/or radiation. The study was initiated in 2015. By October 22, 2020, 220 patients had been included in the CHOCA study; however, only 83 were enrolled at the beginning of 1st-line therapy and had a baseline sample collected before 1st-line therapy. Of these, 62 were eligible for the present study. The remaining were excluded due to the following reasons: prior treatment (surgery and adjuvant therapy) for BTC (N = 15), other cancer at diagnosis or diagnosed within 2 years of BTC diagnosis (N = 4), also included in MICA (N = 1), or patient had combined hepatocellular/cholangiocarcinoma (N =1).

GI1003 study: A phase II trial entitled "Intra-hepatic chemotherapy with oxaliplatin every second week in combination with systemic gemcitabine and capecitabine and in patients with a KRAS-wild type tumor in combination with cetuximab in patient with non-resectable liver metastases from cholangiocarcinoma" [1] (EudraCT: 2010-020188-19, Regional Ethics Committee approval number: H-3-2010-053, the Danish Data Protection Agency approval number HEH.750.24-39). The single-center trial included both cholangiocarcinoma and gallbladder cancer patients, and the inclusion period was from January 2011 to August 2013. In total, 56 patients were included, and baseline samples were available from 50 patients. Of these, 39 were eligible for the present study. The remaining were excluded due to the following reasons: prior treatment (surgery and adjuvant therapy) for BTC (N = 9), other cancer at diagnosis or diagnosed within 2 years of BTC diagnosis (N = 1), or baseline sample collected after initiation of chemotherapy (N = 1).

GI1333 study: A phase II trial entitled: "Randomized 1st-line treatment with gemcitabine, capecitabine, oxaliplatin vs. gemcitabine and cisplatin in patients with cholangiocarcinoma" [2] (EudraCT: 2013-004854-46, Regional Ethics Committee approval number: H-2-2014-026, HEH-

2014-041, the Danish Data Protection Agency approval number: I-Suite 02756BBH). The trial included both cholangiocarcinoma and gallbladder cancer patients. A total of 100 patients were included at 2 sites (Herlev and Vejle Hospital) from July 2014 to November 2017. Eighty-two patients with locally advanced or metastatic BTC were included in the trial at Herlev Hospital. Baseline samples were available from 75 patients, and of these, 57 were eligible for the present study. The remaining were excluded due to the following reasons: prior treatment (surgery and adjuvant therapy) for BTC (N = 14), other cancer at diagnosis or diagnosed within 2 years of BTC diagnosis (N = 1), or baseline sample collected after initiation of chemotherapy (N = 3).

Biliary tract cancer patients from Rigshospitalet, Copenhagen, Denmark

BIOPAC study: The Danish BIOPAC study "BIOmarkers in patients with PAncreatic Cancer (BIOPAC) - can they provide new information of the disease and improve diagnosis and prognosis of the patients?" (ClinicalTrials.gov ID: NCT03311776; www.herlevhospital.dk/BIOPAC/) is a prospective multicenter open cohort study with ongoing enrollment. It has been approved by the Danish Ethics Committee (VEK, j.nr. KA-20060113) and the Danish Data Protection Agency (j.nr. 2012-58-0004; HGH-2015-027; I-Suite j.nr. 03960; and PACTIUS P-2020-834). A subset of the patients was included at the Department of Surgery, Rigshospitalet on suspicion of pancreatic cancer, but were later diagnosed with BTC. As of June 1, 2021, 61 patients with BTC were included in the BIOPAC study prior to a surgical procedure, 48 of whom were eligible for the present study. The remaining patients were excluded due to the following reasons: no available baseline sample (N = 10), prior treatment for BTC (N = 2), or other cancer present at diagnosis or diagnosed within 2 years of BTC diagnosis (N = 1).

Biliary tract cancer patients from Vejle Hospital, Vejle, Denmark

Patients from Vejle hospital were all included in one of the following trials:

GOC-BP study. A phase II trial entitled: "Randomized phase II trial of combination chemotherapy with panitumumab or bevacizumab in patients with inoperable biliary tract cancer without KRAS exon 2 mutations" [3] (Eudract nr. 2010-020385-13, Regional Ethics Committee approval number: S-20100051). Eighty-eight patients with locally advanced or metastatic BTC were randomized to receive chemotherapy (gemcitabine, oxaliplatin, and capecitabine) in combination with either panitumumab or bevacizumab from August 2010 to March 2016. Blood samples were available from 69 patients, and of these 52 were eligible for the present study. The reasons for exclusion were

baseline sample collected after initiation of therapy (N = 7), prior surgery or other treatment for BTC (N = 10). As part of the trial, only EDTA plasma and buffy coat were collected. Therefore, serum samples were not available.

GOX-P study: A phase II trial entitled: "Combined biological treatment and chemotherapy in patients with unresectable cholangiocarcinoma" [4, 5] (Eudract nr. 2008-002367-14, Regional Ethics Committee approval number: S-20080081). The trial was a phase II study in which patients were assigned to treatment based on KRAS exon 2 mutation status. All patients received combination chemotherapy (gemcitabine, oxaliplatin, and capecitabine). Patients with KRAS wild-type tumors also received panitumumab. Patients were included from 2 sites (Vejle, Denmark, Växjö, Sweden). In total, 71 patients were included from October 2008 to 2015, of whom 25 were KRAS exon 2 mutated and 46 were KRAS wild-type. Baseline blood samples were available from 60 patients, and 47 of these patients were eligible for the present study. The reason for exclusion were baseline sample collected after initiation of therapy (N = 4), prior surgery or other treatment for BTC (N = 9). As part of the trial, only EDTA plasma and buffy coat were collected. Therefore, serum samples were not available.

Control cohorts

Controls were included from 1 of the following 3 groups:

MICA Study: The Danish MICA study "New biomarkers in patients referred because of suspected serious illness - are they giving new diagnostic information?" is a prospective open cohort study with ongoing enrollment of patients (older than 18 years) referred to the diagnostic cancer patient pathway at the Diagnostic Outpatient Clinic at Copenhagen University Hospital - Herlev and Gentofte, in the Capital Region of Denmark. The patients are followed from the time of their first visit in the Diagnostic Outpatient Clinic, during evaluation for a cancer diagnosis, and until death. Relevant clinical characteristics of the patients are included in the MICA database. All patients gave written informed consent. The MICA study protocol was approved by the Danish Regional Ethics Committee (H-7-2014-011) and the Danish Data Protection Agency (HEH-2014-105; I-Suite 03330; PACTIUS P-2020-578). In total, 759 patients were included between 2016 and 2019, of whom 8 were diagnosed with BTC (median 12 days (range: 1 – 1178 days) time from date of blood sampling to date of diagnosis. A total of 394 were alive and had no cancer diagnosis after 2 years' follow-up, and they were included as non-cancer controls in the present study. Patients included in the non-cancer control group had the following symptoms at referral: weight loss (50.8%), fatigue

(38.5%), abnormal biochemistry (32.1%), diffuse abdominal pain (23.2%), night sweats (13.8%), pain (9.9%), fever (6.6%), back pain (6.1%), malaise (5.9%). Blood samples were collected at first visit at the Diagnostic Outpatient Clinic.

Benign biliary tract disease (BBTD) cohort: The cohort included 49 patients who had an endoscopic retrograde cholangiopancreatography (ERCP) performed due to BBTD and had been included as controls in a case-control study of post-ERCP pancreatitis at Bispebjerg Hospital, Copenhagen, Denmark [6]. (Regional Ethics Committee approval number: H-15017822). The reasons for ERCP were image-confirmed choledocholithiasis (N = 25), elevated liver enzymes/jaundice (N = 20), or cholangitis (N = 4). Samples were collected during follow-up at a median of 5.2 years (range 1.8 – 7.6 years) after initial ERCP procedure was performed.

Healthy blood donor cohort: Samples were collected from 180 volunteer healthy blood donors at Aalborg University Hospital (Aalborg, Denmark) between January and February 2019.

Protein analysis

Olink immune-Oncology panel

Samples were analyzed for 92 proteins using the proximity extension assay immuno-oncology (I-O) panel from Olink Proteomics, Uppsala, Sweden (www.olink.com). The I-O panel utilizes 92 pairs of antibodies that are linked to DNA oligonucleotides (probes). When a pair of antibodies bind to a target antigen, the probes are brought into proximity. Consequently, the probes are hybridized and extended by a proximity-dependent DNA polymerization event, generating a DNA surrogate marker for the specific antigen (protein). Afterwards, the DNA sequence is quantified by real-time PCR (qPCR). The analyses were performed at BioXpedia, Aarhus, Denmark. BioXpedia was blinded to the study endpoint as no research questions or clinical data were revealed before all samples had been analyzed. For the analyses, serum and EDTA plasma samples were thawed, mixed using a vortex mixer, and centrifuged at 400 g for 1 minute. Then 1 μL of serum or EDTA plasma was transferred to the incubation plate and mixed with the Olink incubation mix and the antibody-probe pairs and analyzed according to the manufacturer's instructions. As recommended by Olink, samples were randomized across assay plates. We also included 2 sample controls (pooled plasma samples), 2 negative controls, and 2 inter-plate control (synthetic samples) on each plate [7]. Additionally, we included 8 bridging samples on all plates used in this project.

Samples were removed if the standard deviation of the internal controls on each plate was above 0.2 NPX, or the deviation from the median value of the controls for each individual sample was above 0.7 NPX. Samples or proteins with more than 10% missing values were removed. Proteins with more than 90% of the value below the limit of detection (LOD) in 1 run were removed.
The samples from the healthy blood donors were analyzed using an older version of the I-O panel (Proseek® Multiplex Immuno-Oncology, v. 953101) in November 2018 and May 2019 as part of a prior study [8]. Later, the manufacturer changed the panel, and the remaining samples were analyzed using a newer version of the panel (Olink Target 96 Immuno-Oncology, v. 953111/v.953112). The samples from patients included in the MICA study were analyzed in October 2019, samples from patients with BTC from Herlev Hospital and patients with BBTD were analyzed in November 2020, and samples from patients included at Vejle Hospital were analyzed in May 2021. A full list of proteins in both panels is available in Supplementary Tables 1 and 2. The following 4 proteins were available only in the old version: IL-21, IL-35, interferon (IFN)-beta, and vascular endothelial growth factor C (VEGFC). These were replaced by the following 4 in the newer version: lymphocyte activation gene 3 protein (LAG3), IL-15, mucin-16 (MUC-16), and killer cell immunoglobulin-like receptor 3DL1 (KIR3DL1). Furthermore, the tumor necrosis factor (TNF) and IFN-gamma assays were markedly improved in the newer version of the panel. Lastly, 5 proteins were removed due to more than 90% of samples being below LOD in at least 1 run (IL-1 alpha, IL-2, IL-13, IL-33, and arginase-1 (ARG1)). Therefore, only 81 proteins were the same in all plates used in the study.

Generation of protein signatures

Samples were normalized for any plate effects using the built-in inter-plate controls according to the manufacturer's recommendations, and the included bridging samples were used for normalization between different version of the I-O panel and plates run at different time points.
Signatures were identified and tested using a multistep strategy. The primary set of protein signatures was identified using the 81 proteins + CA19-9. To test the influence of serum/plasma variabilities on results achieved in validation cohort 1, a second set of signatures was generated using only CA 19-9 + 42 proteins with a constant serum/plasma variation [9].

Step 1: identifying protein signatures in the discovery cohort: The discovery cohort was split randomly into a detection set (2/3 of the data) and a replication set (1/3 of the data). The detection

set was further split randomly into 2 equal parts. A logistic Lasso regression model was fitted on the training set and tested on the test set using the R-package glmnet [10] with alpha = 1 and optimized with the function cv.glmnet using a 10-fold cross validation. This process, including the splitting of the dataset, was repeated 500 times, thus generating 500 different logistic Lasso regression models. For each protein, we calculated the proportion score as the number of times each of the 500 logistic Lasso regression models included that protein as a predictor. The proportion scores were used to generate 21 sets of proteins (signatures). The first set contained proteins with a minimum proportion score of 0 and, thus, contained all the proteins, and the remaining sets were constructed according to incremental steps of the proportion score of 0.05. For sets of proteins that were identical, only the sets with the corresponding highest proportion score were selected, and therefore some of the incremental steps were skipped.

Step 2: fitting signatures on detection set of the discovery cohort: The detection set was split randomly into training and test subsets. For each protein signature, a Ridge regression model was fitted on the training subset of the detection set and tested in the corresponding test subset using the R-package glmnet [10] with alpha = 0 and optimized with the function cv.glmnet using a 10-fold cross validation. The same procedure was carried out with use of the entire detection set as a training set and the entire replication cohort as a test set. For each signature, we fitted a primary model trained to discriminate all BTC from all controls. We also fitted secondary models discriminating between subgroups of BTC (resectable BTC, locally advanced BTC, metastatic BTC, iCC, pCC, dCC, GBC) vs. all controls and all BTC vs. subgroups of controls (patients with BBTD, non-cancer controls, and healthy subjects). Prediction scores were generated for each signature in each cohort. Prediction scores were calculated using NPX and coefficients from each protein as:

$$y = Intercept + NPX.1 * Coefficient.1 + NPX.2 * Coefficient.2 + NPX.X * Coefficient.X$$

$$Prediction\ score = \frac{e^y}{1 + e^y}$$

Step 3: evaluating performance in the discovery cohort: Signature performance was evaluated first in the detection set and afterwards in the replication set. Performance was evaluated using receiver operating characteristic (ROC) curves of prediction scores for each signature and calculated as the area under the ROC curve (AUC). Sensitivity, specificity, positive predictive value and negative predictive value were identified for optimal cut-off calculated using Youden's index

[11]. Likewise, the optimal cut-offs with sensitivity >0.95 and specificity >0.95 were identified. All signatures were tested with and without age and CA19-9 added as a covariates, and the DeLong test [12] was used to compare the AUCs in the generated models. We evaluated each signatures' performance in discriminating all patients with BTC vs. all controls, subgroups of BTC vs. all controls, and all patients with BTC vs. subgroups of controls.

Step 4: evaluating performance in validations cohort 1: Signatures' performance was evaluated in the entire validation cohort 1 using the above-described procedure.

Step 5: evaluating performance in validation cohort 2: The dataset was sent blinded to the bioinformatician who was given no information regarding diagnostic group. The bioinformatician employed the signatures on validation cohort 2 using best point (for all BTC vs. all controls) identified in replication cohort as the threshold for case identification and noted which patients were identified as having BTC. Afterwards, data were unblinded and compared with the prediction. Following this, ROC curves and Youden's index were used to identify optimal threshold for each signature in validation cohort 2.

Supplementary references

[1] Larsen Fo, Hoegdall DTS, Hoegdall E, Nielsen D. Gemcitabine, capecitabine and oxaliplatin with or without cetuximab in advanced biliary tract carcinoma. Acta Oncol 2016;55:382-385.

[2] Markussen A, Jensen LH, Diness LV, Larsen FO. Treatment of patients with advanced biliary tract cancer with either oxaliplatin, gemcitabine, and capecitabine or cisplatin and gemcitabine-a randomized phase II trial. Cancers (Basel) 2020;12:1975.

[3] Amin NEL, Hansen TF, Fernebro E, Ploen J, Eberhard J, Lindebjerg J, et al. Randomized Phase II trial of combination chemotherapy with panitumumab or bevacizumab for patients with inoperable biliary tract cancer without KRAS exon 2 mutations. Int J Cancer 2021;149:119-126.

[4] Jensen LH, Lindebjerg J, Ploen J, Hansen TF, Jakobsen A. Phase II marker-driven trial of panitumumab and chemotherapy in KRAS wild-type biliary tract cancer. Ann Oncol 2012;23:2341-2346.

[5] Jensen L, Andersen R, Byriel L, Fernebro E, Jakobsen A, Lindebjerg J, et al. Phase II study of gemcitabine, oxaliplatin and capecitabine in patients with KRAS exon 2 mutated biliary tract cancers. Acta Oncol 2019;59:1-4.

[6] Leerhøy B, Shabanzadeh DM, Nordholm-Carstensen A, Novovic S, Hansen MB, Jørgensen LN. Pancreatic function following post-endoscopic retrograde cholangiopancreatography pancreatitis: A controlled cohort study with long-term follow-up. United Eur Gastroent J 2018;6:586-594.

[7] Olink User Manual v. 1.9.5: Olink Proteomics, Uppsala Sweden; 2018.

[8] Lindgaard SC, Sztupinszki Z, Maag E, Chen IM, Johansen AZ, Jensen BV, et al. Circulating protein biomarkers for use in pancreatic ductal adenocarcinoma identification. Clinical cancer research : an official journal of the American Association for Cancer Research 2021;27:2592-2603.

[9] Christensen TD, Maag E, Madsen K, Lindgaard SC, Nielsen D, Johansen JS. Determination of temporal reproducibility and variability of cancer biomarkers in serum and EDTA plasma samples using a proximity extension assay. Clin Proteomics 2022;19:39.

[10] Friedman J, Hastie T, Tibshirani R. Regularization paths for generalized linear models via coordinate descent. J Stat Softw 2010;33:1-22.

[11] Ruopp MD, Perkins NJ, Whitcomb BW, Schisterman EF. Youden Index and optimal cut-point estimated from observations affected by a lower limit of detection. Biom J 2008;50:419-430.

[12] DeLong ER, DeLong DM, Clarke-Pearson DL. Comparing the areas under two or more correlated receiver operating characteristic curves: a nonparametric approach. Biometrics 1988;44:837-845.
